# Transcriptome-Based SNP Discovery and Validation in the Hybrid Zone of the Neotropical Annual Fish Genus *Austrolebias*

**DOI:** 10.3390/genes10100789

**Published:** 2019-10-11

**Authors:** Graciela García, Néstor Ríos, Verónica Gutiérrez, Sebastián Serra, Marcelo Loureiro

**Affiliations:** 1 Sección Genética Evolutiva, Facultad de Ciencias, UdelaR, Iguá 4225, Montevideo 11400, Uruguay; nrriosp@gmail.com (N.R.); vgutierrez@fcien.edu.uy (V.G.); serraelbicho@gmail.com (S.S.); 2 Departamento de Ecología y Evolución, Facultad de Ciencias, UdelaR, Iguá 4225, Montevideo 11400, Uruguay; marcnagual@gmail.com; 3 Sección Ictiología, Museo Nacional de Historia Natural, Montevideo 11400, Uruguay

**Keywords:** annual killifish, hybrid zone, *Austrolebias*, transcriptome-based SNP, discovery, validation, morphology, conservation

## Abstract

The genus *Austrolebias* (Cyprinodontiformes: Rivulidae) represents a specious group of taxa following annual life cycles in the neotropical ichthyofauna. They live in temporary ponds and each generation must be completed in a few months, depending on environmental stochasticity. Annual fish survive the dry season through diapausing eggs buried in the substrate of these ponds. A hypothesized bimodal hybrid zone between two taxa of the genus, *A. charrua* and *A. reicherti* from Dos Patos Merin lagoon system, was recently proposed based on genetics and morphological analyses. However, hundreds of additional nuclear molecular markers should be used to strongly support this hypothesized bimodal pattern. In the present paper, we conducted RNA-seq-based sequencing of the transcriptomes from pools of individuals of *A. charrua*, *A. reicherti* and their putative natural hybrids from the previously characterized hybrid zone. As a result, we identified a set of 111,725 SNP (single nucleotide polymorphism) markers, representing presumably fixed allelic differences among the two species. The present study provided the first panel of 106 SNP markers as a single diagnostic multiplex assay and validated their capacity to reconstruct the patterns of the hybrid zone between both taxa. These nuclear markers combined with *Cytb* gene and morphological analyses detected a population structure in which some groups among the hybrid swarms showed different level of introgression towards one or the other parental species according to their geographic distribution. High-quality transcriptomes and a large set of gene-linked SNPs should greatly facilitate functional and population genomics studies in the hybrid zone of these endangered species.

## 1. Introduction

Hybrid zones are remarkable systems for understanding the origin and persistence of species diversity. They constitute reservoirs of polymorphism and are key to the maintenance of biodiversity [1]. Identifying a history of hybridization still leaves the question of how it affects the evolutionary trajectory of lineages [2]. The study of natural hybridization provides insights into the nature of species boundaries and the process of speciation [3,4,5,6].

Given the fact that the neotropical ichthyofauna is one of the most abundant and diverse on Earth, with more than 5600 species [7], it is striking that so few studies have been conducted for natural hybrid zones in this vast region. One of these scarce studies comprises the replicated hybrid zone between *Xiphophorus birchmanni* and *X. malinche* in the Sierra Madre Oriental of eastern Mexico, where natural hybrids were found and characterized [8]. More recently a hybrid zone between two taxa of the South American killifish genus *Austrolebias* (Cyprinodontiformes: Rivulidae): *A. charrua* and *A. reicherti* was characterized for the first time [9]. *Austrolebias* genus represents a specious group of taxa with an annual life cycle among neotropical ichthyofauna, in South America. They live in temporary ponds and each generation must be completed in a few months, depending on environmental stochasticity. Annual fish survive the dry season through diapausing eggs buried in the substrate of these temporary ponds. A spectacular species radiation has been reported for *Austrolebias* from the Pampas [10,11], where high morphological diversity and extensive karyotype divergence have been recorded for this genus [12,13,14]. This burst of speciation process in *Austrolebias* was associated with an unusually large genome size increase within the Aplocheiloidei, with an average DNA content of about 5.95 ± 0.45 picograms per diploid cell (mean C-value of about 2.98 pg) [14]. Additionally, the occurrence of different types of chromosome rearrangements [13,15,16] and a parallele explosive expansion of the transposable elements (TEs) representing more than 45% of the diploid *Austrolebias* genomes [17,18] were reported. These events may have taken place in a vast area of low relief subjected to seasonal flooding which includes lagoons and several rivers that originated during the Quaternary marine transgressions [19,20]. The first characterization of the hybrid zone between the parapatrically distributed *A. charrua* and *A. reicherti* taxa included microsatellite loci, one mitochondrial marker and morphological studies [9]. This hybrid zone spans in a narrow area of approximately 106 km^2^ within the Patos-Merin drainage system (DMS). Previous phylogeographic studies had proposed scenarios of past allopatric fragmentation and range expansion involving secondary contact between both taxa followed by genetic and morphological divergence [13,21]. Moreover, the divergence between *A. charrua* and *A. reicherti* was estimated to have occurred since 100,000 years ago (late Pleistocene). In fact, hybrid zones are traditionally assumed to form via secondary contact after geographic divergence in allopatry [5]. In particular, species in rapidly diversifying adaptive radiations may be particularly prone to hybridization [22,23,24].

In the hybrid zone between *A. charrua* and *A. reicherti*, a hypothetic bimodal pattern was proposed. Hybrid swarms were predominantly individuals genetically similar to one or another of the parental genotypes, with no F1 hybrid progeny. In fact, some of the admixed individuals were morphologically not distinguishable from pure individuals of one or the other parental species [9]. As suggested by Vähä et al. [25] many additional nuclear molecular markers should be used to strongly support this hypothesized bimodal pattern. Therefore, a more comprehensive sampling approach is needed to investigate potential past admixture in areas where the species distributions could have overlapped. Since bidirectional backcrossing involving both parental taxa was detected previously [9], studies including hundreds of nuclear molecular markers are needed to delimit potential genomic regions that have restricted introgression and could be associated to reproductive barriers [26]. Additionally, these markers could contribute to discerning the role of introgression increasing morphological and genetic variability underlying secondary contact.

In this work, we attempted to address the open question about the existence of a possible bimodal pattern and levels of introgression involved in the hybrid zone between *A. charrua* and *A. reicherti*. To accomplish this objective, the sampling area was extended, at the genomic level numerous nuclear molecular markers were obtained and *cytochrome b* (*Cytb*) gene and morphometric analyses were included. To avoid problems with the large genome size of these diploid species and its high level of redundancy, we conducted RNA-Seq based sequencing of the transcriptomes from pools of individuals of *A. charrua*, *A. reicherti* and their putative natural hybrids to obtain more than 100 single nucleotide polymorphisms (SNPs) from expressed sequences. This methodological strategy was proposed by De Wit et al. [27] and it was also implemented in other fish species [28]. High-quality transcriptomes and a large set of gene-linked SNPs should greatly facilitate the functional and population genomics studies in these endangered species.

## 2. Materials and Methods

### 2.1. Re-Mapping of the Hybrid Zone

**Figure 1:**
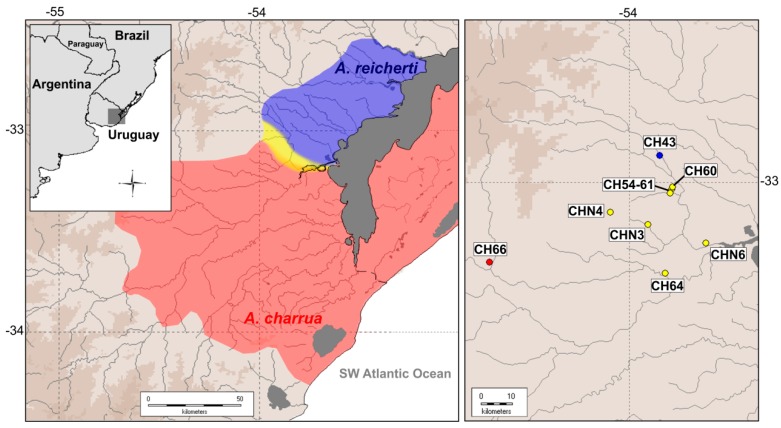
Geographic distribution of taxa in the hybrid zone. *A. charrua* (red area), *A. reicherti* (blue area) and hybrid populations (yellow areas) in the Dos Patos-Merin drainage system (DMS), South America. In the right-hand box, the hybrid ponds in the contact zone between both taxa are indicated.

The present work includes a re-mapping during 2017 and 2018 of the hybrid zone area previously described between *A. charrua* and *A. reicherti* [9]. In 2017, due to flooding, several hybrid ponds (i.e., CH54 and CH61) collapsed in a vast area. However, during 2018 new putatively hybrid ponds were surveyed (CHN3, CHN4, CHN6, CH64) in addition to the previously described ponds CH60, CH54-61, CH62. In total, 8 temporary ponds were sampled: two of parental species *A. charrua* (pond CH66) and *A. reicherti* (pond CH43) and 6 of their putative hybrids detected in geographically intermediate ponds (CH60, CH64, CH54-61, CHN3, CHN4 and CHN6) on the southern and northern margins of the Corrales del Parao stream in the DMS (Figure 1, Table S1). In each pond, specimens of the parental species were easily distinguished on the basis of morphology and geographic distribution through the whole species range. Tissues and voucher specimens are deposited in the Fish Collection (ZVCP) and in the Sección Genética Evolutiva of the Facultad de Ciencias, Universidad de la República, Montevideo, Uruguay. All sampling protocols for this scientific study were approved by the CNEA (Comisión Nacional de Experimentación Animal) of Uruguay (approved protocol number: 240011-001192-16).

### 2.2. Population Genomic Analyses of the Hybrid Zone

#### 2.2.1. Sample Collection for RNA-Seq

Liver and testicle tissues of *A. charrua*, *A. reicherti* and hybrid individuals, were preserved immediately after collection in RNAlater (Sigma-Aldrich, Product No. R0901, Texas, USA) (Table S1). Total RNA was extracted using the RNeasy Universal Tissue Kit (Qiagen, Valencia, CA, USA). The quality and integrity of each RNA was evaluated in a 2% agarose gel and by RIN measurements (RNA integrity number) performed in an Agilent 2100 bioanalyzer (Agilent Technologies, Palo Alto, CA, USA). Pools of high-quality RNA were obtained in order to implement the transcriptome-based SNPs discovery for population analyses. For liver tissue, the RNA pools of *A. reicherti* consisted of 4 males and 8 females, those of *A. charrua* included 6 males and 6 females, and those of hybrids populations consisted of 5 males and 6 females (Tables S1 and S2). For testicles, the RNA pools of *A. reicherti* and *A. charrua* included 4 specimens, whereas that of hybrid populations consisted in 3 individuals. The integrity of each “RNA pool” was determined by RIN measurement as described above. Finally, the 9 “RNA pools” were selected to construct libraries using TruSeq Stranded mRNA LT Sample Prep Kit and sequencing using the mRNA paired-end strategy performed on the Novaseq 6000 platform (MACROGEN, Seoul, Korea), that guarantees more than 120 million reads per sample (Table S2).

#### 2.2.2. Annotation and Mapping of Sequencing Reads

Before the analysis, raw reads were trimmed using Trimmomatic 0.38 [29] by removing adapter sequences and ambiguous nucleotides. Reads with quality scores <20 and length below 30 bp were removed. The resulting high-quality sequences of the 9 transcriptomes were used for the detection of SNPs in two steps: aligning the reads of each individual transcriptome with the reference transcriptome of *A. charrua* [30] using the aligner bowtie 2 v. 2.3.4.1 (Johns Hopkins University, Baltimore, MD, USA) [31], and searching for positions that differ among the different alignments and the reference transcriptome using the software SAMtools and bcftools package (http://www.htslib.org/) [32]. Mapping of reads from each pooled sample to the composite reference assembly sequence was performed with a mismatch cost of 6, a deletion cost of 5 and an insertion cost of 5. A minimum coverage (read depth) ≥10 with a maximum of 8000, was set for each group to assess the quality of reads at positions for SNP detection. Each SNP was annotated against the unpublished genome of *A. charrua* [30].

#### 2.2.3. Validation of Fixed-Allele Interspecific Single Nucleotide Polymorphisms (SNPs)

Since we used pooled samples, we focused particularly on the identification of SNPs with fixed or nearly fixed allelic differences between *A. charrua* and *A. reicherti* (e.g., homozygous ‘C’ in *A. charrua*, homozygous ‘T’ in *A. reicherti* and heterozygous ‘C/T’ in the hybrid population). SNPs that showed the consensus base (100% allele frequency) in one species, and the alternative allele in the other one, with both alleles present in the hybrid population read file (minor allele frequency ≥10%, minimum coverage ≥10), were carried forward as putative fixed-allele diagnostic SNPs.

The “iPLEX Assay Design and Validation” and “iPLEX Genotyping/Allelotyping, Standard Scale” services (Minnesota University, USA) were employed to validate a subset of 130 among the identified SNPs. Only SNPs with at least a 100 bp flanking region on either side of the polymorphic site were selected for the assay design. The final multiplex was tested on a plate including a subset of 94 DNA samples belonged to parental populations of *A. charrua* from pond CH66 (*N* = 10), of *A. reicherti* from CH43 (*N* = 14) and from putative hybrid ponds of CH60 (*N* = 21), CH54-61 (*N* = 12), CHN3 (*N* = 8), CHN4 (*N* = 7), CHN6 (*N* = 12) and CH64 (*N* = 10), (see Table S1). Genomic DNA was isolated from liver tissue (fixed in ethanol 95%) obtained from freshly sacrificed animals by an overexposure to a solution of 1‰ 2-phenoxyethanol (Sigma), using extraction with sodium chloride protein precipitation, followed by ethanol precipitation (modified from Medrano et al. [33]). Selected samples presented a fixed mass of 500 ng.

#### 2.2.4. Statistical Analyses Based on SNP Markers

Population genetic analyses included 94 individuals described in the Section 2.2.3. (see Table S1). The number of alleles, the observed heterozygosity (H_O_), and the expected heterozygosity (He) were calculated for each locus per population and taxon using the GENEPOP 4.2 (Montpellier University, Montpellier, France) [34], ARLEQUIN v. 3.5 software packages [35], and FSTAT v. 2.9.3.2 (University of Lausanne, Lausanne, Switzerland) [36]. GENEPOP 4.2 (Montpellier University, Montpellier, France) [34] was used to perform the exact test for Hardy Weinberg-equilibrium (HWE) by SNP loci (test multi-population) and by population (test multi-locus) using the Markov chain method with 1.000 iterations. The magnitude and sign of departure from HWE at each locus (heterozygote deficit and excess) were analyzed using the intra-population fixation index (F_IS_) [37]. Linkage disequilibrium between loci and deviations from HWE for each locus were tested by a Markov chain method following the algorithm of Guo and Thompson [38] and using the Boferroni correction [39] for multiple comparisons (α = 0.05). All the analyses outlined above were implemented in GENEPOP 4.2 [31].

#### 2.2.5. Population Structure and Patterns of Hybridization Based on SNP Markers

The number of individuals per population used in the structure and patterns of hybridization analyses were described in Section 2.2.3. (see Table S1). For the identification of clusters and graphical representation of the between-group structure, a discriminant analysis of principal components (DAPC) scatter plot was performed using the R package ADEGENET [40]. This analysis allows unraveling the complex population structure in the hybrid zone. This analysis is not based on pre-defined population genetics models and makes no assumptions about HWE or linkage disequilibrium. For this purpose, the number of principal components to be retained was 20, which contain 80% of the cumulative variation of the data.

Considering all 103 polymorphic loci, different hypotheses of grouping as sources of variation were assessed in the analysis of molecular variance (AMOVA). Fst values for pairwise comparisons among the 8 ponds populations and their significant level for genetic differentiation (*p* = 0.05) were obtained performing 1.000 permutations. All these analyses were performed using ARLEQUIN v. 3.5 software package [35].

An analysis of population subdivision and clustering of individual genotypes was implemented with STRUCTURE v. 2.3 [41]. We used the Bayesian method implemented in this program to distinguish hybrids from ‘pure’ species. This method uses a Markov Chain Monte Carlo (MCMC) algorithm to assign individuals (as represented by their multi-loci genotypes) to genetic clusters (K) by minimizing within-group linkage-disequilibrium and simultaneously assuming within-group HWE. We considered 1 to 11 populations (K = 1 to K = 11), two ‘pure’ ones representing the parent species and the remaining six from the contact zone, including 3 other additional K hypotheses. Ten independent runs employing an admixture model were implemented with a burn-in period length of 100,000 iterations, followed by 200,000 MCMC replicates. The consensus result for each K was obtained from independent runs by means of CLUMPAK [42]. The most probable K value was determined using both likelihood and Delta k criteria (Dk [43]) and calculated using STRUCTURE HARVESTER [44].

#### 2.2.6. Estimates of Recent Introgression

The number of individuals per population included in the estimates of recent introgression analysis were described in Section 2.2.3. (see Table S1). In order to assess the pattern and direction of recent hybridization among populations at each site, individuals were classified into genealogical classes using the methods implemented in the NewHybrids v. 1.1 (University of Washington, Seattle, WASH, USA) [45]. This analysis is a model based on probability, which is calculated through MCMC yielding to the posterior probability (qi) of individuals belonging to different genealogical classes. The sensitivity of NewHybrids results was examined with Uniform and Jeffrey prior distributions. Posterior probabilities were evaluated after 200,000 iterations of MCMC, with an initial burn-in of 100,000 steps. As suggested by Vähä et al. [25], a posterior probability >50% was used as a threshold for assigning an individual to a specific class. The default six genealogical classes correspond to (i) pure *A. charrua*, (ii) pure *A. reicherti*, (iii) F1 hybrid, (iv) F2 hybrid, (v) backcross towards *A. charrua* (Bx *charrua*), (vi) backcross towards *A. reicherti* (Bx *reicherti*).

### 2.3. Mitochondrial Sequence Analyses

The analysis based on the *cytochrome b* (*Cytb*) gene included a total of 159 individuals belonging to parental populations of *A. charrua* from pond CH66 (*N* = 16), of *A. reicherti* from CH43 (*N* = 20) and from putative hybrid ponds of CH60 (*N* = 35), CH54-61 (*N* = 21), CHN3 (*N* = 12), CHN4 (*N* = 7), CHN6 (*N* = 37) and CH64 (*N* = 11), (see Table S1). Genomic DNA was isolated from liver tissue fixed in ethanol 95% as described above. A fragment of approximately 700 bp from the *Cytb* gene was amplified using the CB3-H (5′-GGCAAATAGGAARTATCATTC-3′) and Gludg-L (5′-TGACTTGAARAACCAYCGTTG-3′) primers [46]. Polymerase chain reaction (PCR) amplifications were performed in 20 μL reactions containing 1 × Buffer, 1.5 mM MgCl_2_, 0.2 mM each dNTP, 0.5 μM of each primer, 0.5 units of *Taq* DNA polymerase (Invitrogen) and approximately 100 ng of template DNA. Cycling conditions included an initial denaturation of 3 min at 94 °C, followed by four cycles of: 94 °C for 1 min, 42 °C for 1 min, 72 °C for 1 min, followed by 29 cycles of: 94 °C for 1 min, 50 °C for 1 min, 72 °C for 1 min and a final extension of 7 min at 72 °C. PCR products were purified and subsequently sequenced in both strand directions, using the same amplification primers, in a Perkin-Elmer ABI Prism 377 Automated Sequencer (MACROGEN, Seoul, Korea).

Multiple sequence alignment was performed by using the ClustalW tool implemented in MEGA v. 7.0 (Tokyo Metropolitan University, Tokyo, Japan) [47]. Nucleotide composition and substitution patterns were calculated using MEGA and DnaSP v. 5 (University of Barcelona, Barcelona, Spain) [48]. The corrected estimates of pairwise sequence divergence were obtained using the two-parameter algorithm (K2P) of Kimura [49] implemented in MEGA. Within a population, DNA polymorphism was measured calculating the haplotype diversity h [50] (p. 179), and the nucleotide diversity π [50] (p. 257) using DnaSP. Tajima’s test [51] was implemented in DnaSP to test for mutation/drift equilibrium and departure from neutrality.

#### 2.3.1. Population Genetic Structure Based on Cytb Gene

The haplotype network based on *Cytb* sequences was constructed with NETWORK v. 5.0.0.0 (Fluxus Technology Ltd, Suffolk, England) [52]. Genetic structure of *A. charrua*, *A. reicherti* and their putative hybrid populations was assessed using the variance components among hierarchical partitions in the dataset as implemented in the AMOVA analysis [51] using ARLEQUIN v. 3.5 [35]. The Euclidean metric [53] was used to obtain the matrix of pairwise distances. The genetic variation was partitioned into three components: among groups (Φ_CT_), among populations within groups (Φ_SC_), and among individuals within populations (Φ_ST_), disregarding either their original populations or their groups. The eight populations were ascribed to different groups and different grouping hypotheses were tested.

### 2.4. Morpho-Meristic Analyses

Morphological analyses were conducted to confirm the identity of species and their putative hybrids detected by the molecular markers. Specimens analyzed using the morphometric approach comprised 123 males (57 *A. charrua*, 22 *A. reicherti*, and 45 putative hybrids) and 138 females (65 *A. charrua*, 29 *A. reicherti*, and 44 putative hybrids), whereas 23 males and 20 females were included in the general morphological analyses. Specimens were deposited in the Fish Collection (ZVCP) of the Facultad de Ciencias, Universidad de la República, Montevideo, Uruguay.

Morphological variation was assessed using a geometric morphometric analysis of landmark configurations. Shape variation was analyzed by geometric morphometrics using a thin plate spline approach [54]. Fifteen fixed landmark positions were defined according to D’Anatro and Loureiro [55] and obtained from scanned fish images (HP, ScanJet, 5590), digitizing using TPSdig software [56]. Weight matrices of partial warps and the centroid size of specimens were generated using TPSregr [56]. Shape differences among groups were tested by multivariate analysis of covariance (MANCOVA) using the centroid size (the geometric morphometric parameter of body size) as a covariate. Visualization of specimen grouping and the corresponding shape variation were obtained by canonical variate analyses. Due to their pronounced sexual dimorphism, males and females were analyzed separately.

## 3. Results

### 3.1. Transcriptome Sequencing, SNP Detection and Annotation

Table S2 presents the raw data statistics, including the total number of bases, reads, GC (%), Q20 (%), and Q30 (%) calculated for each one of the 9 transcriptomes obtained. The cDNAs were sequenced on Illumina NovaSeq platform that generated a maximum value of 236.6 million paired-end reads for the female liver transcriptome of *A. charrua*, and a minimum for the liver of males from hybrids sample (129.5 million paired-end reads). RNA-Seq data were mapped onto reference transcriptome of *A. charrua* [27]. As a result, of a total number of 275,397 markers, we identified a set of 111,725 SNPs with could be fixed-allelic differences between *A. charrua* and *A. reicherti*. These markers belong to a total of 22,490 contig transcripts.

### 3.2. SNP Validation by the iPLEX Assay Design

Of a subset of the randomly selected 130 SNPs to perform the diagnostic multiplex assay, one failed because of its high dimer potential formation for both forward and reverse primers. Another 5 loci were discarded due to their lower call rates, and 18 SNP loci failed to genotype most of the samples. Finally, the genotype data were generated using a panel of 106 SNP loci, with 44 SNPs called at a 100% success rate, 53 SNP called at 90% and 9 SNP at 70%. These success rates were calculated using 90 of the 94 samples (samples with call rates >70%). Table S3 shows 106 loci analyzed, their annotation, and the fixed-allelic differences between genotypes of *A. charrua*, *A. reicherti* and hybrids according to transcriptomic analysis and posterior genotyping of sub-samples per population. Remarkably, most loci were fixed in one of the parental and were polymorphic in the other parent and in the hybrid population.

### 3.3. Genetic Diversity Parameters Based on 106 SNPs

In order to estimate the genetic diversity parameters, all 106 SNPs were genotyped for polymorphism in 94 samples belonging to parental populations of *A. charrua* (CH66), *A. reicherti* (CH43), and from hybrid populations from ponds CH60, CH54-61, CHN3, CHN4, CHN6, CH64 (Table S1). Table S4 presents the allele frequencies of the 106 loci. Of the successfully genotyped loci, a total of 103 SNPs were found to be polymorphic and 3 were monomorphic. Table 1 shows the expected heterozygosity (He), P-values from HWE deviations and intra-population fixation index (F_IS_) for each locus per population and taxon. Among 103 polymorphic SNPs, expected heterozygosity (He) estimate ranged from 0.386 in the putative hybrid population of CHN3 to 0.243 in the parental pond CH43 of *A. reicherti*. Departures from HWE were detected in markers for some populations (CH43 and CH60) although most of these SNP markers were at equilibrium after Bonferroni correction (*p* = 0.001) (Table S5). The intra-population fixation index (F_IS_) was the highest for all loci in *A. reicherti* (0.141) whereas *A. charrua* from CH66 showed the lowest (−0.338), but none of these values were significant after Bonferroni correction (Table 1). Linkage disequilibrium (LD) after Bonferroni correction (*p* = 0.001) was detected in three putative hybrid populations between some pair of loci as follows: in pond CH60, between SNP_054 vs. SNP_095; in pond CH54-61, between loci SNP_055 vs. SNP_060; and in pond CHN6, between loci SNP_009 vs. SNP_019, and SNP_019 vs. SNP_050.

### 3.4. Patterns of Hybridization and Population Genetics Structure Based on 103 SNPs

AMOVA results in Table 2, a showed that four groups represent the most plausible hypothesis with the largest percentage of variance occurring “within populations”, followed by “among groups”, whereas the “among populations within groups” and “among individuals within populations” showed the lowest values. One group included populations of *A. reicherti* (pond CH43), a second group comprised putative hybrid ponds CH64, CH54-61, CHN3, CHN4 a third group included hybrid ponds CHN6 and CH60, and fourth group was integrated by the population from CH66 of *A. charrua*.

Pairwise Fst values among the 8 populations are presented in Table 3. The highest differentiation values were detected between the populations of *A. reicherti* (CH43) to those of *A. charrua* (CH66), as well as between the first population and populations from ponds CH64, CHN3, CHN4 and CH54-61. On the other hand, the putative hybrid ponds CH60 and CHN6 remained differentiated from *A. charrua*. The lowest Fst values were detected in ponds CH64 and CHN4 in relation to pond CH54-61, as well as between ponds CH60 and CHN6.

**Figure 2:**
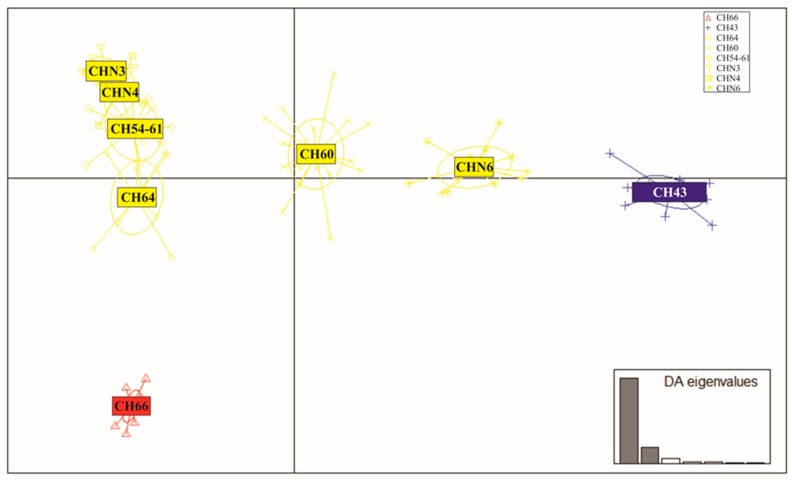
Discriminant analysis of principal components (DAPC) scatter plots [36] based on 103 SNP loci for eight populations of the parental species *A. charrua* and *A. reicherti* and putative hybrid ponds. Populations are shown by colours: *A. charrua* (red), *A. reicherti* (blue), hybrid populations (yellow) and 95% inertia ellipses, squares represent individual genotypes. Axes show the first two discriminant functions, and eigenvalues the genetic information retained by discriminant functions.

DAPC in Figure 2 distinguished both parental species delimited and separated as two distantly clusters while hybrid genotypes from ponds CH60 and CHN6 were an intermediate position. An additional group constituted by ponds CH64, CHN3, CHN4 and CH54-61 appeared spatially segregated in the two axes from the aforementioned three clusters, with pond CH64 being the nearest to parental *A. charrua*.

**Figure 3:**
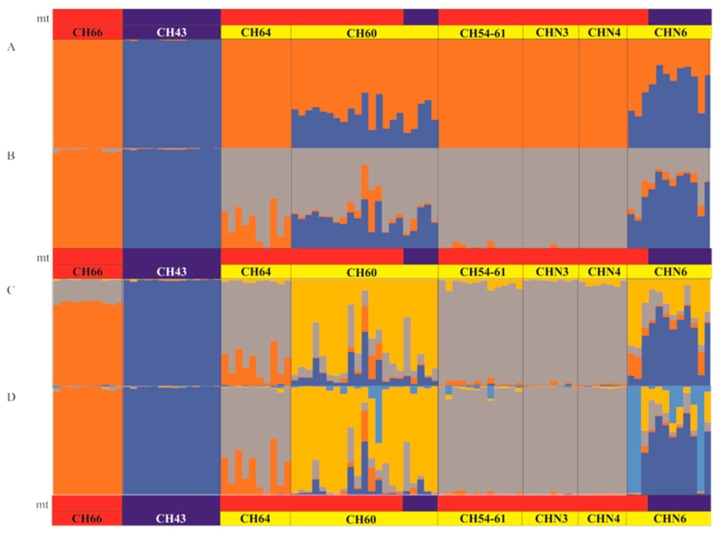
Individual assignment results using STRUCTURE software based on 103 loci. (**A**) K = 2; (**B**) K = 3, (**C**) K = 4 and (**D**) K = 5. Admixture analyses showing the proportion of the genome of each individual originated from *A. charrua* or *A. reicherti*. Each individual is represented as a vertical bar divided into segments representing the proportion of the genome corresponding to *A. charrua* (red) or *A. reicherti* (blue). Populations are labeled above the bar plots; mt bars: represent the *Cytb* haplotype assignation of each individual to *A. charrua* (red) or *A. reicherti* (blue) species.

The Bayesian-based clustering analysis using 103 nuclear markers performed in STRUCTURE showed that the optimal value of K was 2 (K = 2; ΔK = 2810.80; Mean Ln(PX|K) = −7315.82), corresponding to the ‘pure’ populations of *A. charrua* and *A. reicherti* respectively (Figure 3A). This analysis identified only the ponds CH60 and CHN6 as putative hybrids. The remaining candidate hybrid ponds (CH54-61, CHN3, CHN4, CH64) shared their major genomic proportion with *A. charrua*. Interestingly, other structures indicated as likely values were accessed with K = 3 to K = 5 (K = 3, ΔK = 0.90; Mean Ln(PX|K) = −7088.30; K = 4, ΔK = 0.08; Mean Ln(PX|K) = −6991.90; K = 5, ΔK = 43.94; Mean Ln(PX|K) = −6886.96), (Figure 3B–D). Under the hypothesized structure K = 3 (Figure 3B), all the aforementioned ponds appeared with different proportion of admixture from both parental taxa but they were all identified as putative hybrid ponds. In contrast, the hypothesized structures K = 4 and K = 5 denoted the existence of four genomic differentiated clusters as follows: each parental species as a separated cluster; putative hybrid ponds (CH60 and CHN6); an additional group constituted by ponds CH54-61, CHN3, CHN4 and CH64. This last group represented a differentiated cluster showing basal admixture but in particular pond CH64 shared a considerable proportion of the *A. charrua* genome. Another interesting feature from the hypothesized structure K = 5 was the detection of a sub-structure within CHN6, perhaps representing introgressed hybrid swarms toward the *A. reicherti* genome, as suggested by *Cytb* analyses. Thus, Figure 3 also includes the mitochondrial ancestry of individuals in order to clarify the direction of the introgression in each population. Ponds CH54-61, CHN3, CHN4 and CH64 shared mitochondrial lineage with *A. charrua*. The other hybrid ponds presented different proportions of both mitochondrial lineages: pond CH60 shared the most common haplotypes with *A. charrua*, whereas pond CHN6 did so with *A. reicherti*.

**Figure 4:**
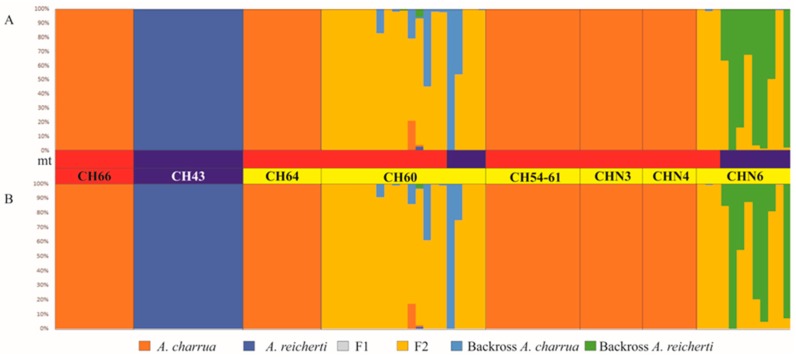
Posterior probabilities of the ancestral genotype class estimated with NewHybrids. (**A**) Uniform prior assumption. (**B**) Jeffrey prior assumption. Each individual is represented as a vertical bar divided into six segments. Each color indicates the posterior probability of an individual assignment to pure *A. reicherti* (blue), pure *A. charrua* (red), F1 (grey), F2 (yelow), and first generation backcross of a F1 hybrid with a pure *A. reicherti* (BC1R, green) or with a pure *A. charrua* (BC1C, sky). Populations are labeled above the bar plots. mt bars: represent the *Cytb* haplotype assignation of each individual to *A. charrua* (red) or *A. reicherti* (blue) species.

NewHybrids software was used to detect recent hybridization events with a threshold value of 0.5, which showed an efficiency to identify hybrids similar to that of the STRUCTURE analysis. Both Jeffrey and Uniform prior distribution showed the same inference about hybrid categories (Figure 4A, B). Only ponds CH60 and CHN6 appeared as hybrid populations assigned predominately to F2 ancestry class and only some individuals correspond to backcrosses toward *A. charrua* in the first pond and toward *A. reicherti* in the last one. Interestingly, neither of the prior assumptions in NewHybrids detected F1 individuals. Under this analysis, ponds CH54-61, CHN3, CHN4 and CH64 were assigned to the *A. charrua*-like genotype. Among the hybrid individuals, a uniform prior assumption (Figure 4A) displayed high probabilities (>50%) of belonging to different classes distributed as follows: 52.86% to *A. charrua* parental; 37.14% to F2; 14% backcrossed toward *A. charrua*; 86% backcrossed toward *A. reicherti*. Jeffrey prior assumption (Figure 4B) displayed high probabilities (>50%) of belonging to *A. charrua* parental (52.86%); F2 (40.00%); backcrossed toward *A. charrua* (1.43%) and toward *A. reicherti* (5.71%).

### 3.5. Mitochondrial Sequence Variation and Population Analyses

A matrix of 795-bp of *Cytb* sequences from 159 individuals belonging to 8 populations from the three taxa was analyzed in the present work. All sequences were grouped into 29 haplotypes (H1–H29). Haplotype assignation per sample is listed in Table S1. These new sequences have been deposited in the GenBank (Accession numbers: MN149431-MN149518 and MN219411-MN219434).

Table 4 presents the estimate of *Cytb* polymorphism in each of 8 populations in the hybrid zone between *A. charrua* and *A. reicherti* from DMS in South America. The highest intra-population sequence distance using K2P model was observed in the hybrid population of pond CHN6 (3.8%) and the lowest (0.1%), in the parental *A. reicherti* (from pond CH43). The lowest inter-population pairwise K2P sequence divergence was detected between CHN3 and CH64 whereas the highest values were observed inpond CH43 of *A. reicherti* in relation to *A. charrua* from pond CH66, and the remaining ponds CH64, CHN3, CHN4, CH54-61 (Table S6).

**Figure 5:**
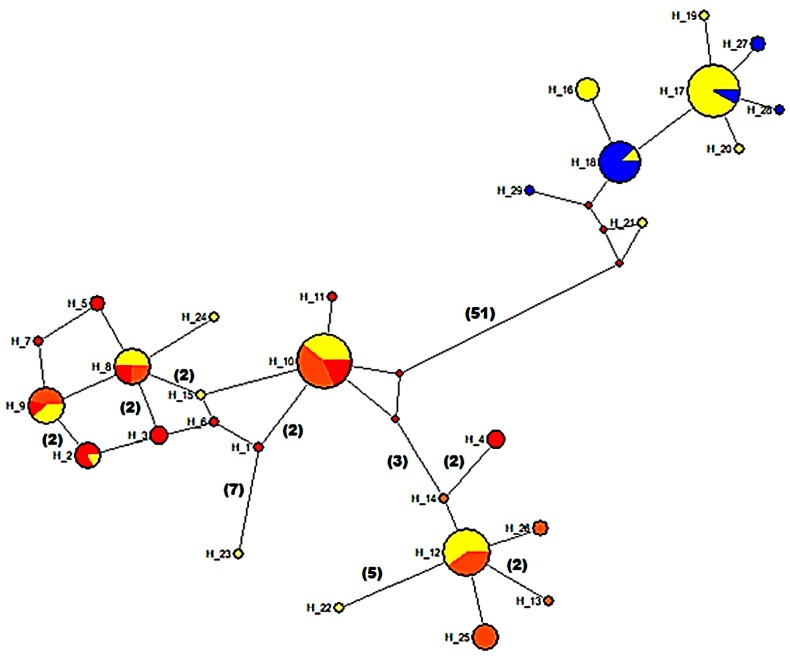
Haplotype network of 29 halotypes based on mitochondrial *Cytb* gene in 159 individuals of *A. charrua* (**red**), *A. reicherti* (**blue**) and hybrid populations from ponds CH60 and CHN6 (**yellow**). Populations from ponds CHN3, CHN4, CH64 and CH54-61 (**orange**). The circles are proportional to the haplotype frequency. Step-mutations values higher than one are between brackets.

The median-joining analysis resulted in a haplotype network with two major clades connected by 51 step mutations (Figure 5). One of these major clades presented two minor clades connected by a central and most frequent haplotype H_10 formed by samples from ponds CH64, CHN3, CH54-61, and hybrid ponds CH60 and CHN6. One of these minor clades was formed by haplotypes mainly belonging to hybrid ponds (CH60-CHN6), as well as from ponds CH64, CHN3, CH54-61 and CHN4. The second minor clade additionally included some haplotypes of *A. charrua* from pond CH66 connected by a one- or two-step mutation and showing reticulation in relation to the other haplotypes from this pond. On the other hand, the other major clade comprises the two most frequent haplotypes. One of them, the H_18 haplotype includes mainly samples from *A. reicherti* and some samples from the hybrid pond CHN6. Conversely, haplotype H_17 includes samples mostly from hybrid ponds CHN6 and CH60 and only two samples from *A. reicherti*.

AMOVA results showed that three groups were the most plausible population structure hypothesis with the largest percentage of variance detected among groups (Table 2, b). Under this hypothesis, one group comprised *A. reicherti* from pond CH43 of and hybrid pond CHN6; a second group included *A. charrua* from pond CH66; a third group comprised the putative hybrid ponds CH54_61, CH60, CHN3, CHN4 and CH64.

Table 5 presents pairwise Fst values among 8 populations. The highest differentiation (Fst > 0.50) was detected among populations of *A. reicherti* vs. all remaining ponds excepting, the hybrid population from pond CHN6.

### 3.6. Morphology

Multivariate analysis of covariance in males generally resulted in significant differences (Wilks lambda = 0.07, F = 2.41; *p* = 0.0000). In paired comparisons, after the Bonferroni correction of p values, only *A. charrua* exhibited significant differences with *A. reicherti* (Wilks Lamda = 0.35, F = 3.42 *p* = 0.0001), CH60 (Wilks lamda = 0.31, F = 3.29; *p* = 0.0003), and CHN6 (Wilks lambda = 0.24, F = 4.17, *p* = 0.000). In females, differences among all groups also resulted statistically significant (Wilks lamda = 0.08, F = 2.61, *p* = 0.0000). As observed in males, the only paired comparison with significant differences corresponded to *A. charrua* in relation to *A. reicherti* (Wilks lamda = 0.37, F = 3.94, *p* = 0.0000), to CH60 (Wilks lambda = 0.34, F = 3.19, *p* = 0.0003), and to CHN6 (Wilks lamda = 0.21, F = 5.77, *p* = 0.0000).

**Figure 6:**
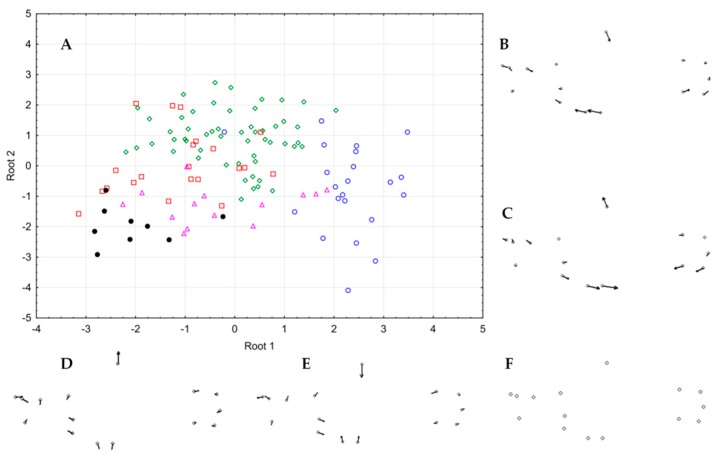
**A**. Scatter plots from the canonical variate analysis (CVA) of the geometrical morphometric analyses of males of the *A. charrua* (green diamonds), *A. reicherti* (blue open dots), CHN6 (black closed dots), CH60 (pink triangles), CH54-CH61, CH64, CHN3, CHN4 (red squares); **B.** Shape deformation along root 2 positive values; **C.** Shape deformation along root 2 negative values; **D.** Shape deformation along root 1 negative values; **E.** Shape deformation along root 1 positive values; **F.** consensus of landmarks configuration. All deformations multiplied by a factor of three for a better visualization of shape change.

**Figure 7:**
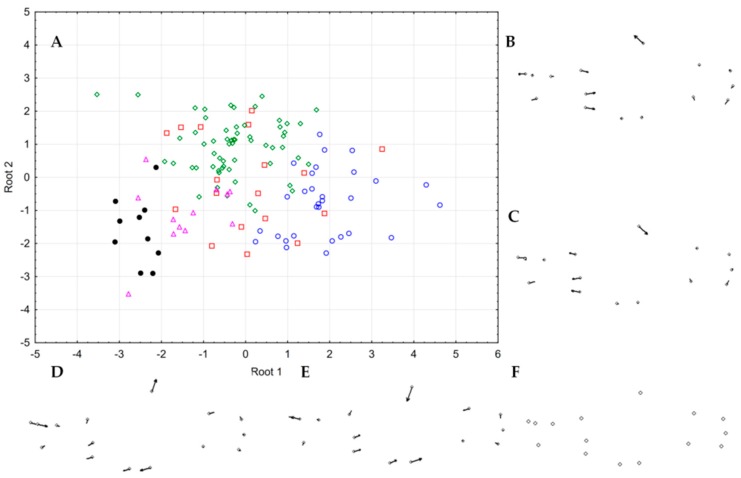
**A.** Scatter plots from the canonical variate analysis (CVA) of the geometrical morphometric analyses of females of the *A. charrua* (green diamonds), *A. reicherti* (blue open dots), CHN6 (black closed dots), CH60 (pink triangles), CH54-CH61, CH64, CHN3, CHN4 (red squares); **B.** Shape deformation along root 2 positive values; **C.** Shape deformation along root 2 negative values; **D.** Shape deformation along root 1 negative values; **E.** Shape deformation along root 1 positive values; **F.** consensus of landmarks configuration. All deformations multiplied by a factor of three for a better visualization of shape change.

*Austrolebias charrua* males differentiated from *A. reicherti* along canonical root 1 (41%), by a deeper and smaller head, and from CH60 and CHN6 along Root 2 (28%) by a shallower body and an anterior displacement of the anal fin origin (Figure 6). *Austrolebias charrua* females differentiated from *A. reicherti* along canonical root 1 (37%) by a deeper body; and, from CH60 and CHN6 along root 2 (26%) by a deeper body and larger head (Figure 7).

## 4. Discussion

### 4.1. Geographic Delimitation of the Present Hybrid Zone

The present work represents a robust contribution to the analysis of the hybrid zone between populations of *A. charrua* and *A. reicherti* inhabiting the biosphere reserve and Ramsar sites in South America (Figure 1). Our study reports a re-sampling of the area, the genotyping of hundreds of novel nuclear SNP markers corresponding to particular genes, and mitochondrial *Cytb* and morphological analyses from previously reported and new hybrid localities. The previously recorded hybrid zone comprised a narrow area of approximately 106 km^2^ across a spatial transect which involved five geographically intermediate ponds (CH54, CH53, CH60, CH61 and CH62). This hybrid zone was only on the southern margin of the Corrales del Parao stream, a tributary of the Cebollatí River basin in the DMS [9]. After 14 years of fieldwork and laboratory analysis using other genetic markers, the present hybrid zone remains localized and spatially reduced to two patches on both margins of the Corrales del Parao stream: pond CH60 on the southern margin of the Corrales del Parao stream and a new hybrid pond, CHN6, on the northern margin (Figure 1). Pond CH60 is surrounded by other ponds (CH54-61, CH64, CHN3 and CHN4) in which individuals showed intergradation of the morphological and pigmentation patterns towards *A. charrua* [9]. By contrast, in the northern margin, the neighboring ponds of pond CHN6 are inhabited by individuals morphologically *reicherti*-like [9].

### 4.2. Structure of the Hybrid Zone

STRUCTURE results with K = 2, allowed us to support a probable hypothesis of a bimodal structure for this hybrid zone (Figure 3A). This result was concordant with the previously hypothesized bimodal hybrid zone between *A. charrua* and *A. reicherti* based on seven microsatellite loci and one mitochondrial marker [9]. However, the present multilocus genotypic analyses from DAPC (Figure 2), STRUCTURE (K = 3–K = 5) (Figure 4B–D), AMOVA (Table 2, a) and morphological analyses suggested a more complex structure in this hybrid zone. All combined methods allowed unmasking the existence of different groups among the hybrid swarms involved in the contact area of these taxa.

One of these groups comprised hybrid ponds CH60 and CHN6, located on both margins of the Corrales del Parao stream. These ponds presented high levels of heterozygosity and *Cytb* polymorphism in relation to parental taxa. Additionally, departure from HWE in pond CH60 and the LD detected between some pairs of loci in both ponds could be consistent with their hybrid swarm status. On the other hand, Newhybrid analysis detected that most individuals belonged to F2 ancestry class and some of them corresponded to backcross with *A. charrua* in pond CH60 whereas almost half of specimens in pond CHN6 evidenced backcross with *A. reicherti*. All these new analyses corroborated the existence of bidirectional backcrossing between both taxa as was previously reported [9].

Other putative hybrid ponds (CH54-61, CHN3, CHN4 and CH64) are located in the Southern of the Corrales del Parao stream and around pond CH60. Remarkably, these ponds showed the highest expected heterozygosity values. According to Twyford and Ennos [57] when introgression occurs, high level of allele diversity in the vicinity of the hybrid swarm can be expected, as detected in the present study. These ponds represent a differentiated group of hybrid ancestry showing a high level of introgression towards the *A. charrua* genome, as supported by *Cytb* analysis, Newhybrid and STRUCTURE (K = 2) results. On the other hand, *Cytb* results, AMOVA, DAPC, STRUCTURE (from K = 3 to K = 5), and morphological analyses could suggest that the combination of hybrid genotypes and introgression have generated in this group a new genomic entity, which is different from *A. charrua* and from the remaining hybrid pondsIn fact, the pairwise Fst comparisons based on the *Cytb* gene showed a high level of differentiation between this group of populations and *A. charrua*. In contrast a lowest differentiation in relation to the hybrid pond of CH60 was observed. Additionally, it cannot be ruled out in this group the possible occurrence of past introgression events (or re-colonization by *A. charrua*-like populations), which could explain their closeness with this parental taxon in some of the implemented analyses.

Therefore, in present paper, the ponds CH60 and CHN6 could represent the start of hybridization and introgression processes toward one or another parental species, whereas in the remaining ponds CH54-61, CHN3, CHN4 and CH64 a higher introgression towards the *A. charrua* genome would be observed.

Moreover, DAPC and multivariate analysis of covariance in males and females were consistent in detecting statistically significant differences between *A. charrua* in relation to *A. reicherti* and from individuals of hybrid ponds CH60 and CHN6. In fact, morphological analysis showed evidence of subtle changes in some body characters in males and females of the CH60 and CHN6 ponds in relation to the parental *A. charrua*. This fact could indicate divergent selection acting in such environments and/or the occurrence of possible character displacement among these taxa. Following Brown and Wilson [58] in the area of overlapping, where the two species occur together, the populations are more divergent and easily distinguished, i.e., they “displace” one another in one or more characters to avoid competition. As many authors have proposed character displacement in both ecological and reproductive traits thereby alters the targets of sexual selection within species and the selective context in which sexual signals are expressed and perceived [59,60], and could be related to reinforcement. The existence of reinforcement in this hybrid zone has been previously proposed based on strong female mating choice system and assortative mating previously described between the parental taxa [61,62]. Therefore, the aforementioned morphological character changes reported in present paper could be related to or be indicative of the reinforcement in this hybrid zone.

### 4.3. Dynamics Underlying Patterns and Processes in the Hybrid Zone

Movement and stability may be plausible scenarios for the hybrid zone because contact among populations of these Austrolebias taxa inhabiting in para/sympatry depend on seasonal flooding, when ponds may collapse in an extensive area. These unstable and heterogeneous environments represent major challenges where the genomic/phenotypic plasticity are required to overcome selective constraints in each temporal pond. Following Abbott [63], hybrid populations could exhibit lower fitness relative to parents as in tension hybrid zones. Conversely, they could present variable fitness as may be the case with hybrid swarms comprising a wide range of early and later generation hybrids, as observed in the present *Austrolebias* hybrid zone. In this last case, hybrids of different generations seem to be segregating in more or less nearby ponds depending on rainfall levels and their potential connection.

Moreover, previous analyses suggested the long term existence of the hybrid zone between *A. charrua* and *A. reicherti* which has been subject to spatial/temporal movements from the North to South and in the reverse direction, reaching the lowland region near the Atlantic Ocean, in accordance with geological and climatic changes in the recent past [9]. As a result of many successive contacts between both taxa subtle mechanisms of reproductive isolation involving the aforementioned reinforcement may have been acquired suggesting that some level of pre- and/or post-zygotic reproductive isolation is operating to prevent the complete merging of these two species.

The extensive geological instability of this area could explain the separation of the present hybrid zone in two patches (ponds CH60 and CHN6) through the actual geographical barrier constituted by the Corrales del Parao stream. Interestingly, network *Cytb* analysis showed that some haplotypes H_2, H_9, H_10, H_12, H_16 located on the southern margin of this stream were shared by individuals from both ponds CH60 and CHN6 as well as by individuals of *A. charrua* (CH66) and the remaining ponds (CH64, Ch54-61, CHN3, CHN4). In contrast, the H_17 haplotype situated on the northern margin of this stream was shared by individuals belonging to hybrids ponds CHN6 and CH60 and *A. reicherti*, whereas H_16 was common among specimens from CHN6 and CH60. This fact constitutes robust support for the recent past movement of the hybrid zone and the separation of the patches through the Corrales del Parao stream. This hybrid zone movement could be associated with major basin transformation. In this sense, some authors have reported the capture of alluvial fans located in the South as part of the Cebollatí river in the DMS drainage, during the Holocene, as a clear demonstration of its recent activity and the extensive reorganization of this basin [19,64].

### 4.4. Further Prospects and Conservation Remarks

Mainly due to giant genome size [14,17,18], *Austrolebias* genomics, remains extremely challenging. The availability and high-quality transcriptomes and a large set of gene-linked SNPs, should greatly facilitate functional and population genomics studies in these species allowing the identification of traits and processes under selection during introgressive hybridization. At the same time, in present work the 106 validated SNP markers revealed the existence of a peculiar structure that does not support the previously hypothesized bimodal pattern for the hybrid zone between *A. charrua* and *A. reicherti*. In fact, all hybrid populations analyzed showed a different degree of introgression towards one or another parental species. Moreover, the nuclear dataset and the morphological analyses showed that hybrid populations are different entities from both parents. Since hybrids’ mosaic genomes aggregating genes from different parental lineages can create novel phenotypes [65], hybridization provides a mechanism to release populations from adaptive limits [66]. Moreover, Hamilton and Miller [66] suggest that genetic variation that persists within natural hybrids may have conservation value, and natural introgression between sympatric or parapatric sister species could be considered an in situ conservation strategy [67]. Therefore, the present results reinforce the importance of the natural hybridization and introgression in the analyzed hybrid zone between *A. charrua* and *A. reicherti* to preserve in situ biodiversity by increasing the observed phenotypic variation in each temporal pond where these two endangered killifish species inhabit.

## 5. Conclusions

The present work represents a robust contribution to the analysis of the hybrid zone between *A. charrua* and *A. reicherti* inhabiting the biosphere reserve and Ramsar sites in South America. The present hybrid zone remains localized and spatially reduced in two patches in both margins of the Corrales del Parao stream in the DMS basin. High-quality transcriptomes and a large set of gene-linked SNPs should greatly facilitate functional and population genomics studies in these endangered species. The analyses performed, such as population genomic based on 106 validated SNPs loci, the *Cytb* gene and morphology detected a complex population structure in the hybrid zone between *A. charrua* and *A. reicherti* that was not concordant with a typical bimodal pattern. All combined methods allowed unmasking the existence of different groups among the hybrid swarms involved in the contact area, with different levels of introgression towards one or other parental taxa, according to their geographic distribution. Moreover, these analyses corroborated the existence of bidirectional backcrossing between both species as was previously reported [9]. Finally, the present results reinforce the importance of natural hybridization and introgression in the hybrid zone between *A. charrua* and *A. reicherti* to preserve in situ biodiversity of these endangered taxa.

## Figures and Tables

**Table 1:** Diversity based on 103 single nucleotide polymorphism (SNP) loci in 8 populations in the hybrid zone between *A. charrua* and *A. reicherti* from DMS in South America.

Population	N	He (s.d)	*p*	F_IS_ (s.d)
CH64	10	0.373 (0.130)	0.753	−0.023 (0.651)
CH66	10	0.280 (0.186)	0.237	−0.338 (0.996)
CH43	14	0.243 (0.141)	0.001	0.141 (0.073)
CH60	21	0.331 (0.159)	0.000	−0.100 (0.944)
CH54-61	12	0.340 (0.161)	0.460	0.019 (0.456)
CHN3	8	0.386 (0.146)	0.751	−0.029 (0.621)
CHN4	7	0.349 (0.153)	0.981	−0.019 (0.617)
CHN6	12	0.303 (0.143)	0.007	−0.033 (0.671)

N: number of individual per population; He: expected heterozygosity per population; *p*: departures from Hardy-Weinberg equilibrium (HWE); F_IS_: inbreeding coefficient. The values were not significant after sequential Bonferroni correction *p* < 0.001. (s.d: standard deviation between brackets).

**Table 2:** Hierarchical analysis of molecular variance (AMOVA) of 8 populations belonging to the two parental species (*A. charrua* and *A. reicherti*) and six putative hybrid populations from a contact zone in the DMS in South America (Figure 1, Table S1).

Hypothesis	Source of Variation	*df*	Sum of Squares	Variance Components	Percentage of Variation	Φ Statistics
a	Among groups	3	217.221	1.41953 Va	26.25	Φ_CT_ = −0.263 (0.001)
	Among population within groups	4	39.714	0.29998 Vb	5.55	Φ_SC_ = 0.075 (0.000)
	Among individuals within populations	86	304.043	−0.15252 Vc	−2.82	Φ_IS_ = −0.041 (0.885)
	Within populations	94	361.000	3.84043 Vd	71.02	Φ_IT_ = 0.290 (0.000)
b	Among groups	2	1423.097	15.54290 Va	65.39	Φ_CT_ = 0.653 (0.010)
	Among population within groups	5	144.291	1.24626 Vb	5.24	Φ_SC_ = 0.151 (0.000)
	Within populations	147	1026.096	6.98024 Vc	29.37	Φ_ST_ = 0.706 (0.000)

For each molecular marker, the more plausible grouping hypothesis among all tested, was: a) based on 103 SNPs, four groups of populations as follows: one group constituted by CH43 of *A. reicherti*; a second group integrated by putative hybrids from ponds CH54-61, CHN3, CHN4 and CH64; a third group including putative hybrids from ponds CHN6 and CH60; a fourth group integrated by ponds CH66; b) based on *Cytb* separating three groups of samples as follows: one group integrated by CH43 of *A. reicherti* and the hybrid pond CHN6; a second group including the pond CH66 of *A. charrua*; a third group comprising the putative hybrid ponds CH54-61, CH60, CHN3, CHN4 and CH64. *p*-values are given in parentheses.

**Table 3:** Pairwise Fst comparisons based on 103 SNP loci in 8 populations from the hybrid zone of DMS in South America. Significant values (*p* = 0.05) are in bold.

		1	2	3	4	5	6	7	8
1	CH66	0.00000							
2	CH43	**0.639**	0.00000						
3	CH54-61	**0.240**	**0.515**	0.00000					
4	CHN4	**0.385**	**0.605**	**0.009**	0.00000				
5	CHN3	**0.401**	**0.613**	**0.068**	**0.100**	0.00000			
6	CH64	**0.149**	**0.524**	**0.040**	**0.145**	**0.161**	0.00000		
7	CH60	**0.310**	**0.255**	**0.127**	**0.173**	**0.241**	**0.169**	0.00000	
8	CHN6	**0.357**	**0.152**	**0.207**	**0.267**	**0.304**	**0.225**	**0.016**	0.00000

**Table 4:** Comparative estimates of *Cytb* DNA polymorphism among 8 populations in the hybrid zone between *A. charrua* and *A. reicherti* from DMS in South America.

Population	N	HpN	h (s.d)	*π* (s.d)	K2P	*D*
CH64	11	4	0.745	0.002	0.002	0.628
			(0.098)	(0.001)	(0.001)	*p* > 0.10
CH66	16	7	0.858	0.006 (0.001)	0.007	1.024
			(0.057)		(0.002)	(NS)
CH43	20	6	0.579	0.001	0.001	−1.332
			(0.015)	(0.000)	(0.001)	*p* > 0.10
CH60	35	8	0.800	0.004	0.028	1.164
			(0.001)	(0.000)	(0.004)	(NS)
CH54-61	21	8	0.865	0.005	0.005	0.357
			(0.002)	(0.000)	(0.001)	*p* > 0.10
CHN3	12	6	0.873	0.003	0.002	1.803
			(0.071)	(0.000)	(0.001)	(NS)
CHN4	7	4	0.810	0,005	0.006	0.885
			(0.130)	(0.001)	(0.002)	*p* > 0.10
CHN6	37	13	0.701	0.032	0.038	0.759
			(0.005)	(0.006)	(0.005)	*p* > 0.10

N: number of individual per population; HpN: number of haplotypes; h: haplotype diversity [50]; *π*: nucleotide diversity [50]; K2P: intraspecific pairwise Kimura two parameters distance [49]; *D*: neutrality test [51]; (s.d: standard deviation between brackets). Statistical significance: *p* < 0.05.

**Table 5:** Pairwise comparisons based on 29 haplotypes of the *Cytb* gene in 8 populations from DMS in South America. Significant values (*p* = 0.05) are in bold.

		1	2	3	4	5	6	7	8
1	CH66	0.00000							
2	CH43	**0.96138**	0.00000						
3	CHN6	**0.62306**	**0.17907**	0.00000					
4	CH54-61	**0.61678**	**0.96075**	**0.62750**	0.00000				
5	CH60	**0.29516**	**0.73899**	**0.41500**	**0.11801**	0.00000			
6	CH64	**0.61543**	**0.98576**	**0.59722**	**0.40308**	**0.08874**	0.00000		
7	CHN3	**0.60930**	**0.98489**	**0.59850**	**0.42342**	**0.09494**	-0.06163	0.00000	
8	CHN4	**0.48559**	**0.96341**	**0.55235**	0.03462	0.05959	**0.42159**	**0.41856**	0.00000

## Supplementary Materials

The following are available online at https://www.mdpi.com/2073-4425/10/10/789/s1: Table S1: List of samples used in this work, Table S2: Transcriptome statistics, Table S3: Validated 106 loci, Table S4: Allele frequencies per population, Table S5: Departure from HWE_in 8 populations, Table S6: Pairwise interpopulation K2P distance based on the *Cytb* dataset.
