# Supplementary material for: Transcriptome-Based SNP Discovery and Validation in the Hybrid Zone of the Neotropical Annual Fish Genus Austrolebias
Source: Genes (Basel). 2019 Oct 11;10(10):789. doi: 10.3390/genes10100789 (PMC6826752; doi:10.3390/genes10100789)
Supplement: Supplementary file 1 [file genes-10-00789-s001.zip › genes-572550-supplementary-proof/Table S1 List of samples.docx]

**Table S1**

List of samples used in this work. Species, localities, geographic coordinates, type of analyses (m = morphological, lots number of the Vertebrate Collection International Code ZVC-P); G = Genomics (*****=Transcriptomes,**^+^**=SNP genotypes, Cytb), ID (Number of Evolutionary Genetics Laboratory Code), mitochondrial Cytb haplotype numbers and GenBank accession numbers of individuals analyzed belonging to 8 populations in DMS from South America.

| **Species** | **Locality** | **Coordinates** | **M** | **G** | **ID** | **Haplotypes** | **Gen Bank**  **Access Number** |
| --- | --- | --- | --- | --- | --- | --- | --- |
| *Austrolebias charrua* | **Treinta y Tres Department,**  **Uruguay** |  |  |  |  |  |  |
|  | Treinta y Tres city (CH66) | -33,226001  -54,396402 | 14480 | G | 4033***^+^**, 4034,  4035***^+^**, 4036***^+^** 4037***^+^**, 4038***^+^**  4039*****, 4040***** 4041, 4042  4043***^+^**,4044***^+^** 4045***^+^**, 4046***^+^** 4047***^+^**, 4048***** | H_1, H_2  H_3  H_3, H_4  H_5  H_2, H_3  H_2, H_6  H_4  H_7,H_2 | MK818277, MK818278 MK818279, MK818280 MK818281, MK818282 MK818283, MK818284 MK818285, MK818286  MK818287, MK818288 MK818289, MK818290 MK818291, MK818292 |
| *Austrolebias reicherti* |  |  |  |  |  |  |  |
|  | **Treinta y Tres Department, Uruguay** |  |  |  |  |  |  |
|  | Road 91, Arrozal Treinta y Tres (CH43) | -32,922928  -53,913816 | 4363 6529 | G | 4008**^+^**,4009***^+^,**  4010***^+^**, 4011**^+^** 4012***^+^**,4013**^+^** 4014*****, 4015 4016*****, 4017***^+^** 4018, 4019  4020***^+^**,4021***^+^** 4022***^+^**, 4023***^+^**  4024*****, 4025***^+^** 4026***^+^**, 4027***^+^** | H_18  H_18  H_27,H_18  H_18, H_17  H_18  H_18, H_28  H_18, H_17  H_18  H_18, H_29  H_27, H_18 | MK818346, MK818347, MK818348 , MK818349, MK818350, MK818351, MK818352, MK818353, MK818354 ,MK818355, MK818356, MK818357, MK818358, MK818359, MK818360, MK818361, MK818362, MK818363, MK818364, MK818365 |
| *Austrolebias* hybrid ponds | **Treinta y Tres Department, Uruguay** |  |  |  |  |  |  |
|  | Road 17, Cañada Chica (CH64) | -33,256779  -53,896987 | 6516 | G | 4088**^+^**,4089**^+^**  4090**^+^**,4091**^+^**  4092**^+^**, 4093**^+^** 4094**^+^**, 4095**^+^**  4097, 4098**^+^** 4099**^+^** | H_8, H_9  H_10  H_10,H_9  H_8, H_10  H_8, H_11  H_10 | MK818311, MK818312 MK818313, MK818314  MK818315, MK818316 MK818317, MK818318 MK818319 , MK818320 MK818321 |
|  |  |  |  |  |  |  |  |
|  | Road 91, S of Corrales del Parao (CH60)  S of Corrales del Parao  (CHN3)  S of Corrales del Parao  (CHN4)  N of Corrales del Parao (CHN6)  Road 91, S of Corrales del Parao (CH54-CH61) | -33,012287  -53,876931  -33.118640°  -53.949041°  -33.083571°  -54.054577°  -33.171099°  -53.781939°  -33,018131°  -53,879357°  -33,029929°  -53,882981° | 6515  14237  14236  14233  4175  6511 | G  G  G  G  G | 4029,4030  4120**^+^**,4121**^+^**  4122**^+^**,4123  4124**^+^**,4125**^+^**  4126**^+^**,4127**^+^**  4128**^+^**,4129**^+^**  4130**^+^**,4131  4132,4133  4134,4135  4136,4137  4138**^+^**,4139  4140,4141**^+^**  4142,4143**^+^**  4144**^+^**,4145**^+^**  4146, 4147**^+^**  4148^+^,4149**^+^**  4150**^+^**,4151**^+^**  4100**^+^**  4101**^+^**,4102**^+^**  4103**^+^**,4104  4105,4106**^+^**  4107**^+^**,4108**^+^**  4109,4110**^+^**  4111**^+^**,4112  4113**^+^**,4114**^+^**  4115**^+^**,4116**^+^**  4117**^+^**,4118**^+^**  4119**^+^**  4152,4153**^+^**  4154,4155**^+^**  4156**^+^**,4157**^+^**  4158**^+^**,4159  4160,4161  4162,4163  4164,4165  4166,4167**^+^**  4168,4169  4170,4171  4172**^+^**,4173**^+^**  4174**^+^**,4175**^+^**  4176,4177  4178**^+^**,4179  4180,4181**^+^**  4182,4183  4184,4185  4186,4187  4188  4031*****,4032*****  4068*****,4069**^+^**  4070***^+^**,4071**^+^**  4072*,4073  4074,4075**^+^**  4076***^+^**,4077  4078***^+^**,4079*****  4081***^+^**,4082***^+^**  4083***^+^**,4084  4085**^+^**,4086***^+^**  4087**^+^** | H_12  H_9, H_10  H_10  H_9,H_12  H_8, H_10  H_10,H_16 H_8, H_10 H_16, H_10 H_12,H_17  H_12  H_12, H_8  H_10  H_15,H_17 H_9,H_8  H_16,H_10 H_17,H_9  H_8, H_12  H_10  H_10  H_10, H_8  H_9  H_9,H_10  H_9,H_8  H_10  H_9, H_12  H_13,H_12  H_9, H_14  H_12  H_18,H_17  H_18, H_19  H_17  H_8, H_17  H_22, H_17  H_17  H_12,H_17  H_12,H_17  H_17, H_23  H_17, H_2  H_10, H_20  H_17, H_12  H_12, H-17  H_9, H_16  H_17, H_21  H_16, H_17  H_17  H_17  H_17  H_24, H_10  H_8, H_25  H_12, H_25  H_26, H_12  H_12, H_25  H_26, H_10  H_25, H_10  H_10, H_12  H_25, H_10  H_10, H_12  H_25 | MN219411, MN219412  MN149450, MN149451  MN149452, MN149453  MN149454, MN149455  MN149456, MN149457  MN149458, MN149476  MN149459, MN149460  MN149477, MN149461  MN149462, MN149478  MN149463, MN149464  MN149465, MN149466  MN149467, MN149468  MN149469, MN149479  MN149470, MN149471  MN149480, MN149472  MN149481, MN149473  MN149474, MN149475  MN219434  MN149431, MN149432  MN149433,MN149434  MN149435,MN149436  MN149437,MN149438  MN149439,MN149440  MN149441,MN149442  MN149443,MN149444  MN149445,MN149446  MN149447, MN149448  MN149449  MN149482, MN149483  MN149484,MN149485  MN149486, MN149487  MN149509, MN149488  MN149510, MN149489  MN149490, MN149491  MN149511, MN149492  MN149512, MN149493 MN149494, MN149513  MN149495, MN149514  MN149515, MN149496  MN149497, MN149516  MN149517, MN149498  MN149518, MN149499  MN149500, MN149501  MN149502, MN149503  MN149504, MN149505  MN149506, MN149507  MN149508  MN219413, MN219414  MN219415, MN219416  MN219417, MN219418  MN219419, MN219420  MN219421, MN219422  MN219423, MN219424  MN219425, MN219426  MN219427, MN219428  MN219429, MN219430  MN219431, MN219432  MN219433 |

The individuals 4144, 4145 and 4147 were not included in the population genomic analyses, due to the lower number of genotyped loci in them.
