# Supplementary material for: Transcriptome-Based SNP Discovery and Validation in the Hybrid Zone of the Neotropical Annual Fish Genus Austrolebias
Source: Genes (Basel). 2019 Oct 11;10(10):789. doi: 10.3390/genes10100789 (PMC6826752; doi:10.3390/genes10100789)
Supplement: Supplementary file 1 [file genes-10-00789-s001.zip › genes-572550-supplementary-proof/Table S2 Raw data Transcriptomes.docx]

**Table S2**

Statistics of the transcriptomes. The total number of bases, reads, GC (%), Q20 (%), and Q30 (%) are calculated for the 9 Transcriptomes. Sample codes: ARE_ *A. reicherti*; ACH_ *A. charrua*; AHY; *Austrolebias* hybrids; H: female; M: male; H: liver; T: Testicle. (Number of samples in pools of each transcriptome in brackets).

| **Sample ID** | **Total read bases (bp)** | **Total reads** | **GC(%)** | **AT(%)** | **Q20(%)** | **Q30(%)** |
| --- | --- | --- | --- | --- | --- | --- |
| AREMH(4) | 13,889,508,890 | 137,519,890 | 50.287 | 49.71 | 97.994 | 94.406 |
| AREHH(8) | 14,256,686,310 | 141,155,310 | 49.287 | 50.71 | 97.983 | 94.558 |
| AHYMH(5) | 13,082,540,100 | 129,530,100 | 49.911 | 50.09 | 98.035 | 94.424 |
| AHYHH(6) | 17,937,517,584 | 177,599,184 | 48.598 | 51.4 | 97.937 | 94.534 |
| ACHMH(6) | 15,428,377,614 | 152,756,214 | 49.254 | 50.75 | 97.501 | 93.304 |
| ACHHH(6) | 23,895,573,234 | 236,589,834 | 48.900 | 51.1 | 97.710 | 93.985 |
| AREMT(4) | 14,161,510,172 | 140,212,972 | 46.637 | 53.36 | 94.394 | 88.181 |
| AHYMT(3) | 16,677,828,010 | 165,127,010 | 50.921 | 49.08 | 97.542 | 93.489 |
| ACHMT(4) | 15,602,277,192 | 154,477,992 | 49.521 | 50.48 | 96.107 | 91.022 |

- Sample ID: Pool sample name.
- Total read bases: Total number of bases sequenced.
- Total reads : Total number of reads. For Illumina paired-end sequencing, this value refers to the sum of read 1 and read 2.
- GC(%) : GC content.
- AT(%) : AT content.
- Q20(%) : Ratio of bases that have phred quality score of over 20.
- Q30(%) : Ratio of bases that have phred quality score of over 30.
