# Supplementary material for: Transcriptome-Based SNP Discovery and Validation in the Hybrid Zone of the Neotropical Annual Fish Genus Austrolebias
Source: Genes (Basel). 2019 Oct 11;10(10):789. doi: 10.3390/genes10100789 (PMC6826752; doi:10.3390/genes10100789)
Supplement: Supplementary file 1 [file genes-10-00789-s001.zip › genes-572550-supplementary-proof/Table S3 selected 106 loci.docx]

**Table S3**

Discovery and selection of 106 loci to genotyping in multiplex assays. Number of each locus; Expressed contig according to the reference transcriptome [30]; SNP position; Annotation; Allelic variants detected by transcriptomic analysis in *A. charrua*, *A. reicherti*, and hybrids; observed allelic frequencies in all genotyped samples.

| **Locus** | **Expressed Contig** | **Position** | **Annotation** | ***A. charrua*** | ***A. reicherti*** | **Hybrids** | **Observed Allelic frequencies all samples** | |
| --- | --- | --- | --- | --- | --- | --- | --- | --- |
| SNP_001 | ACHA01MRNA0000013 | 648 | Glutamate O-methyltransferase | AC | A | AC | C:14.4% | A:85.6% |
| SNP_002 | ACHA01MRNA0000017 | 1042 | Septin-2B | AC | C | CA | C:72.8% | A:27.2% |
| SNP_003 | ACHA01MRNA0000048 | 405 | Multiple RNA-binding domain-containing protein1 | GA | G | GA | G:91.7% | A:8.3% |
| SNP_004 | ACHA01MRNA0000056 | 483 | Mitogen-activated proteinkinase12 | T | C | TC | C:0.0% | T:100.0% |
| SNP_005 | ACHA01MRNA0000122 | 468 | Complement C3 | TC | T | TC | C:12.3% | T:87.7% |
| SNP_006 | ACHA01MRNA0000215 | 582 | Dihydropyrimidine dehydrogenase [NADP(+)] | G | A | GA | G:56.5% | A:43.5% |
| SNP_007 | ACHA01MRNA0000413 | 1143 | Glycogen phosphorylase (liver form) | G | GC | GC | C:15.9% | G:84.1% |
| SNP_008 | ACHA01MRNA0000555 | 315 | 60S ribosomal protein L14-1 | T | CT | TC | T:94.8% | C:5.2% |
| SNP_009 | ACHA01MRNA0000606 | 921 | Heat shock cognate 70kDa protein | GA | G | GA | G:77.5% | A:22.5% |
| SNP_010 | ACHA01MRNA0000629 | 513 | Ceruloplasmin | CT | T | CT | C:43.9% | T:56.1% |
| SNP_011 | ACHA01MRNA0000717 | 381 | Probable mitochondrial phosphate carrier protein | TG | GT | GT | G:67.2% | T:32.8% |
| SNP_012 | ACHA01MRNA0000730 | 240 | Purple acid phosphatase 17 | C | T | CT | T:30.0% | C:70.0% |
| SNP_013 | ACHA01MRNA0001126 | 354 | Cerebellin-1 | T | C | TC | C:16.7% | T:83.3% |
| SNP_014 | ACHA01MRNA0002014 | 417 | 60S ribosomal protein L10 | TC | T | TC | C:25.8% | T:74.2% |
| SNP_015 | ACHA01MRNA0002052 | 447 | Coagulation factor XI | T | TC | TC | C:38.1% | T:61.9% |
| SNP_016 | ACHA01MRNA0002243 | 613 | Elongation factor1-gamma | A | GA | GA | G:29.9% | A:70.1% |
| SNP_017 | ACHA01MRNA0002760 | 274 | 40S ribosomal protein S8-A | A | CA | A | C:60.0% | A:40.0% |
| SNP_018 | ACHA01MRNA0002788 | 635 | Apolipoprotein Eb | GC | G | GC | G:77.8% | C:22.2% |
| SNP_019 | ACHA01MRNA0002935 | 168 | Alpha-2-HS-glycoprotein | G | AG | GA | G:75.8% | A:24.2% |
| SNP_020 | ACHA01MRNA0002960 | 297 | Pigment epithelium-derived factor | T | G | TG | G:31.7% | T:68.3% |
| SNP_021 | ACHA01MRNA0003042 | 366 | Protein disulfide-isomerase | CT | C | CT | T:8.3% | C:91.7% |
| SNP_022 | ACHA01MRNA0003117 | 1407 | Ceruloplasmin | C | T | CT | C:46.1% | T:53.9% |
| SNP_023 | ACHA01MRNA0003225 | 289 | Complement C3 | A | G | AG | G:28.5% | A:71.5% |
| SNP_024 | ACHA01MRNA0003557 | 869 | Complement factor B | T | A | TA | A:81.3% | T:18.8% |
| SNP_025 | ACHA01MRNA0005638 | 336 | 60S ribosomal protein L12-A>lcl\|P0CT84.1RecName:Full=60SribosomalproteinL12-B | C | T | CT | C:81.5% | T:18.5% |
| SNP_026 | ACHA01MRNA0005799 | 675 | Ribosome biogenesis protein NSA2 homolog | CT | C | CT | T:12.4% | C:87.6% |
| SNP_027 | ACHA01MRNA0005894 | 399 | 40S ribosomal protein S16 | C | CT | C | C:97.2% | T:2.8% |
| SNP_028 | ACHA01MRNA0006255 | 903 | Catalase | G | GA | GA | A:80.6% | G:19.4% |
| SNP_029 | ACHA01MRNA0006540 | 157 | ATP synthase F(0) complex subunit C2, mitochondrial | GA | A | AG | A:81.3% | G:18.8% |
| SNP_030 | ACHA01MRNA0006805 | 466 | 60SribosomalproteinL7a-1>lcl\|P0DKK7.1RecName:Full=60SribosomalproteinL7a-2 | A | GA | A | A:98.1% | G:1.9% |
| SNP_031 | ACHA01MRNA0006806 | 101 | Coagulation factor IX | C | GC | C | G:33.9% | C:66.1% |
| SNP_032 | ACHA01MRNA0006811 | 279 | Chymotrypsin-like elastase family member 2B | C | AC | CA | C:95.6% | A:4.4% |
| SNP_033 | ACHA01MRNA0006837 | 171 | Histone H3.3 | A | G | AG | A:76.8% | G:23.2% |
| SNP_034 | ACHA01MRNA0007271 | 1233 | GTPase-activating protein | A | GA | AG | G:23.6% | A:76.4% |
| SNP_035 | ACHA01MRNA0007323 | 1047 | Serpin B8 | A | G | AG | G:32.9% | A:67.1% |
| SNP_036 | ACHA01MRNA0007402 | 615 | Phosphoenolpyruvate carboxykinase, cytosolic [GTP] | AG | A | AG | A:98.3% | G:1.7% |
| SNP_037 | ACHA01MRNA0007770 | 378 | Microsomal triglyceride transfer protein large subunit | GC | C | GC | G:72.5% | C:27.5% |
| SNP_038 | ACHA01MRNA0008912 | 294 | 40S ribosomal protein S3 | T | TA | T | T:93.9% | A:6.1% |
| SNP_039 | ACHA01MRNA0008934 | 171 | Peptidyl-prolyl cis-trans isomerase C | T | CT | CT | C:52.8% | T:47.2% |
| SNP_040 | ACHA01MRNA0009009 | 1143 | Glycogen phosphorylase (liver form) | G | GC | GC | T:17.2% | C:82.8% |
| SNP_041 | ACHA01MRNA0009201 | 1047 | Collagen alpha-1(I) chain | T | GT | TG | G:13.6% | T:86.4% |
| SNP_042 | ACHA01MRNA0009373 | 516 | Alpha-1-antiproteinase 2 | T | CT | T | C:21.8% | T:78.2% |
| SNP_043 | ACHA01MRNA0009565 | 150 | Complement factor B | A | GA | AG | A:61.2% | G:38.8% |
| SNP_044 | ACHA01MRNA0009901 | 426 | Inositol oxygenase | TC | C | CT | C:66.7% | T:33.3% |
| SNP_045 | ACHA01MRNA0009912 | 1089 | T-complex protein 1 subunit alpha | G | GA | GA | G:89.4% | A:10.6% |
| SNP_046 | ACHA01MRNA0009942 | 1368 | Complement component C8 beta chain | C | CT | CT | C:90.0% | T:10.0% |
| SNP_047 | ACHA01MRNA0009943 | 1236 | Complement component C8 alpha chain | G | CG | G | C:22.8% | G:77.2% |
| SNP_048 | ACHA01MRNA0010000 | 546 | Mitochondrial tricarboxylate transporter 1 | C | CT | CT | C:48.3% | T:51.7% |
| SNP_049 | ACHA01MRNA0010445 | 222 | Insulin-like growth factor-binding protein 4 | G | T | TG | G:67.2% | T:32.8% |
| SNP_050 | ACHA01MRNA0010558 | 387 | Elongation of very long chain fatty acids protein 5 | T | CT | T | T:75.6% | C:24.4% |
| SNP_051 | ACHA01MRNA0010766 | 530 | Protein AMBP | G | AG | GA | G:67.2% | A:32.8% |
| SNP_052 | ACHA01MRNA0011634 | 1218 | Nucleolar protein 56 | G | A | GA | A:31.0% | G:69.0% |
| SNP_053 | ACHA01MRNA0011678 | 1032 | Glutamate dehydrogenase, mitochondrial | T | CT | CT | T:51.1% | C:48.9% |
| SNP_054 | ACHA01MRNA0012159 | 432 | Leukocyte elastase inhibitor A | T | CT | TC | C:41.7% | T:58.3% |
| SNP_055 | ACHA01MRNA0012264 | 1227 | Tubulin beta-1 chain | A | C | AC | C:60.0% | A:40.0% |
| SNP_056 | ACHA01MRNA0013107 | 1177 | Fibrinogen alpha chain | AG | A | AG | A:54.5% | G:45.5% |
| SNP_057 | ACHA01MRNA0013656 | 295 | S-methylmethionine-homocysteine S-methyltransferase BHMT2 | T | C | TC | C:35.1% | T:64.9% |
| SNP_058 | ACHA01MRNA0013750 | 162 | Hemopexin | AG | A | AG | A:97.7% | G:2.3% |
| SNP_059 | ACHA01MRNA0014049 | 396 | Peroxiredoxin-1 | TA | T | TA | A:9.8% | T:90.2% |
| SNP_060 | ACHA01MRNA0014604 | 867 | Dolichyl-diphosphooligosaccharide-protein glycosyltransferase subunit STT3A | AG | A | AG | G:42.5% | A:57.5% |
| SNP_061 | ACHA01MRNA0016359 | 13 | Protein-glutamine gamma-glutamyltransferase 2 | CT | C | CT | T:7.2% | C:92.8% |
| SNP_062 | ACHA01MRNA0017332 | 826 | Angiotensinogen | C | CG | CG | G:29.4% | C:70.6% |
| SNP_063 | ACHA01MRNA0017476 | 513 | 60S ribosomal protein L10a | GA | G | GA | G:83.3% | A:16.7% |
| SNP_064 | ACHA01MRNA0017622 | 935 | Properdin | CA | C | CA | C:75.9% | A:24.1% |
| SNP_065 | ACHA01MRNA0017809 | 105 | Inactive squalene synthase 2 | GT | G | GT | G:29.3% | T:70.7% |
| SNP_066 | ACHA01MRNA0018205 | 162 | Selenoprotein Pb | AG | A | AG | A:88.5% | G:11.5% |
| SNP_067 | ACHA01MRNA0019140 | 465 | Tubulin alpha-1C chain | G | GA | GA | G:93.3% | A:6.7% |
| SNP_068 | ACHA01MRNA0019365 | 1326 | 78 kDa glucose-regulated protein | AC | A | AC | C:30.3% | A:69.7% |
| SNP_069 | ACHA01MRNA0019420 | 317 | Complement factor H-related protein 3 | A | TA | TA | A:0.0% | T:100.0% |
| SNP_070 | ACHA01MRNA0020144 | 1752 | Collagen alpha-2(I) chain | C | CT | CT | C:66.5% | T:33.5% |
| SNP_071 | ACHA01MRNA0020162 | 108 | Protein leg1a | G | GA | GA | G:80.0% | A:20.0% |
| SNP_072 | ACHA01MRNA0020437 | 759 | 60S ribosomal protein L3 | T | TC | TC | T:81.6% | C:18.4% |
| SNP_073 | ACHA01MRNA0020669 | 226 | 40S ribosomal protein S3 | T | TC | TC | T:48.3% | C:51.7% |
| SNP_074 | ACHA01MRNA0020752 | 488 | Cyclin-G1 | A | AG | AG | A:92.1% | G:7.9% |
| SNP_075 | ACHA01MRNA0020767 | 987 | Hydroxymethylglutaryl-CoA synthase, cytoplasmic | C | CT | CT | T:12.8% | C:87.2% |
| SNP_076 | ACHA01MRNA0021775 | 1698 | Eukaryotic translation initiation factor 3 subunit L | T | TC | TC | C:9.4% | T:90.6% |
| SNP_077 | ACHA01MRNA0022167 | 1625 | Complement factor H | TC | T | CT | T:95.0% | C:5.0% |
| SNP_078 | ACHA01MRNA0022434 | 1039 | Synaptonemal complex protein 1 | CG | C | CG | C:89.4% | G:10.6% |
| SNP_079 | ACHA01MRNA0023081 | 435 | Cathepsin L1 | CT | C | CT | T:23.9% | C:76.1% |
| SNP_080 | ACHA01MRNA0023361 | 273 | Nascent polypeptide-associated complex subunit beta | T | TG | TG | G:26.7% | T:73.3% |
| SNP_081 | ACHA01MRNA0024161 | 339 | Estradiol 17 beta-dehydrogenase 5 | TC | T | TC | C:23.9% | T:76.1% |
| SNP_082 | ACHA01MRNA0024439 | 206 | Lactose-binding lectin l-2 | G | GT | GT | G:99.4% | T:0.6% |
| SNP_083 | ACHA01MRNA0025722 | 258 | Diablo homolog, mitochondrial | A | AG | AG | A:88.1% | G:11.9% |
| SNP_084 | ACHA01MRNA0026430 | 522 | Glyoxylate reductase | TG | T | TG | G:41.6% | T:58.4% |
| SNP_085 | ACHA01MRNA0027530 | 255 | 60S ribosomal protein L13 | CG | C | CG | G:27.8% | C:72.2% |
| SNP_086 | ACHA01MRNA0030300 | 162 | Eukaryotic translation initiation factor 3 subunit I | T/C | T | TC | C:63.9% | T:36.1% |
| SNP_087 | ACHA01MRNA0030797 | 342 | Venom prothrombin activator trocarin-D | G | GA | GA | G:86.0% | A:14.0% |
| SNP_088 | ACHA01MRNA0031394 | 297 | 40S ribosomal protein S18 | CT | C | CT | T:10.1% | C:89.9% |
| SNP_089 | ACHA01MRNA0031398 | 1569 | Eukaryotic translation initiation factor 3 subunit D | CT | C | CT | T:10.0% | C:90.0% |
| SNP_090 | ACHA01MRNA0031841 | 134 | Kininogen | TC | T | TC | T:85.6% | C:14.4% |
| SNP_091 | ACHA01MRNA0031997 | 624 | Guanine nucleotide-binding protein subunit beta-2-like 1 | GA | G | GA | A:28.7% | G:71.3% |
| SNP_092 | ACHA01MRNA0032329 | 303 | Ubiquitin-40S ribosomal protein S27a | CT | C | CT | C:85.1% | T:14.9% |
| SNP_093 | ACHA01MRNA0032732 | 279 | Fructose-bisphosphate aldolase C | GA | G | GA | A:20.6% | G:79.4% |
| SNP_094 | ACHA01MRNA0032931 | 160 | Fetuin-B | CT | C | CT | C:75.0% | T:25.0% |
| SNP_095 | ACHA01MRNA0032998 | 1625 | Inter-alpha-trypsin inhibitor heavy chain H4 | G | GA | GA | G:54.6% | A:45.4% |
| SNP_096 | ACHA01MRNA0033212 | 1239 | T-complex protein 1 subunit beta | GA | G | GA | A:23.9% | G:76.1% |
| SNP_097 | ACHA01MRNA0033256 | 1229 | Carboxypeptidase A2 | T | TC | TC | C:28.1% | T:71.9% |
| SNP_098 | ACHA01MRNA0034113 | 1458 | Lanosterol 14-alpha demethylase erg11 | CT | C | CT | C:89.0% | T:11.0% |
| SNP_099 | ACHA01MRNA0034258 | 216 | Chymotrypsin B | C | CT | CT | T:0.0% | C:100.0% |
| SNP_100 | ACHA01MRNA0034302 | 2107 | Inter-alpha-trypsin inhibitor heavy chain H4 | C/A | C | CA | C:65.9% | A:34.1% |
| SNP_101 | ACHA01MRNA0034424 | 792 | Peroxidasin-like protein | GC | G | GC | C:16.7% | G:83.3% |
| SNP_102 | ACHA01MRNA0036093 | 909 | Keratin, type II cytoskeletal 8 | A | AC | AC | C:16.5% | A:83.5% |
| SNP_103 | ACHA01MRNA0036718 | 561 | Dolichyl-diphosphooligosaccharide-protein glycosyltransferase subunit 2 | GT | G | GT | G:72.4% | T:27.6% |
| SNP_104 | ACHA01MRNA0037663 | 726 | Glucokinase | C | CT | CT | C:79.2% | T:20.8% |
| SNP_105 | ACHA01MRNA0039596 | 1437 | Liver carboxylesterase 2 | TC | T | TC | C:3.4% | T:96.6% |
| SNP_106 | ACHA01MRNA0040823 | 234 | Ependymin | AG | A | AG | A:96.5% | G:3.5% |
