# Supplementary material for: Transcriptome-Based SNP Discovery and Validation in the Hybrid Zone of the Neotropical Annual Fish Genus Austrolebias
Source: Genes (Basel). 2019 Oct 11;10(10):789. doi: 10.3390/genes10100789 (PMC6826752; doi:10.3390/genes10100789)
Supplement: Supplementary file 1 [file genes-10-00789-s001.zip › genes-572550-supplementary-proof/Table S4 Allele frequencies.docx]

Table S4

Allele frequencies for the 106 genotyped loci in 94 individuals from 8 populations. Codes of populations are as in Figure 1.

|  |  | **CH64** | **CH66** | **CH43** | **CH60** | **CH54-61** | **CHN3** | **CHN4** | **CHN6** |
| --- | --- | --- | --- | --- | --- | --- | --- | --- | --- |
| SNP_ 001 | N | 10 | 10 | 14 | 19 | 12 | 8 | 7 | 12 |
|  | p: 1 | 0,750 | 0,950 | 1,000 | 0,921 | 0,708 | 0,688 | 0,786 | 0,917 |
|  | p: 2 | 0,250 | 0,050 | 0,000 | 0,079 | 0,292 | 0,313 | 0,214 | 0,083 |
| SNP_ 002 | N | 10 | 10 | 14 | 21 | 12 | 8 | 7 | 12 |
|  | p: 1 | 0,650 | 0,600 | 0,000 | 0,190 | 0,458 | 0,250 | 0,071 | 0,042 |
|  | p: 2 | 0,350 | 0,400 | 1,000 | 0,810 | 0,542 | 0,750 | 0,929 | 0,958 |
| SNP_003 | N | 10 | 10 | 14 | 20 | 12 | 8 | 7 | 12 |
|  | p: 1 | 0,150 | 0,000 | 0,000 | 0,075 | 0,125 | 0,188 | 0,214 | 0,000 |
|  | p: 3 | 0,850 | 1,000 | 1,000 | 0,925 | 0,875 | 0,813 | 0,786 | 1,000 |
| SNP_004 | N | 10 | 10 | 14 | 18 | 12 | 8 | 7 | 12 |
|  | p: 4 | 1,000 | 1,000 | 1,000 | 1,000 | 1,000 | 1,000 | 1,000 | 1,000 |
|  |  |  |  |  |  |  |  |  |  |
| SNP_005 | N | 10 | 10 | 0 | 11 | 12 | 8 | 7 | 7 |
|  | p: 2 | 0,050 | 0,100 | NA | 0,273 | 0,208 | 0,063 | 0,071 | 0,000 |
|  | p: 4 | 0,950 | 0,900 | NA | 0,727 | 0,792 | 0,938 | 0,929 | 1,000 |
| SNP_006 | N | 9 | 10 | 14 | 17 | 11 | 8 | 5 | 11 |
|  | p: 1 | 0,222 | 0,000 | 1,000 | 0,471 | 0,091 | 0,125 | 0,100 | 0,955 |
|  | p: 3 | 0,778 | 1,000 | 0,000 | 0,529 | 0,909 | 0,875 | 0,900 | 0,045 |
| SNP_007 | N | 9 | 10 | 13 | 18 | 11 | 7 | 6 | 11 |
|  | p: 2 | 0,222 | 0,150 | 0,077 | 0,250 | 0,136 | 0,000 | 0,167 | 0,182 |
|  | p: 3 | 0,778 | 0,850 | 0,923 | 0,750 | 0,864 | 1,000 | 0,833 | 0,818 |
| SNP_008 | N | 9 | 10 | 14 | 17 | 10 | 8 | 7 | 12 |
|  | p: 2 | 0,111 | 0,050 | 0,000 | 0,029 | 0,050 | 0,188 | 0,071 | 0,000 |
|  | p: 4 | 0,889 | 0,950 | 1,000 | 0,971 | 0,950 | 0,813 | 0,929 | 1,000 |
| SNP_009 | N | 10 | 10 | 14 | 20 | 12 | 8 | 6 | 12 |
|  | p: 1 | 0,250 | 0,000 | 0,000 | 0,375 | 0,417 | 0,063 | 0,333 | 0,250 |
|  | p: 3 | 0,750 | 1,000 | 1,000 | 0,625 | 0,583 | 0,938 | 0,667 | 0,750 |
| SNP_010 | N | 10 | 10 | 14 | 18 | 12 | 8 | 7 | 12 |
|  | p: 2 | 0,700 | 0,850 | 0,000 | 0,417 | 0,500 | 0,563 | 0,429 | 0,250 |
|  | p: 4 | 0,300 | 0,150 | 1,000 | 0,583 | 0,500 | 0,438 | 0,571 | 0,750 |
| SNP_011 | N | 10 | 10 | 14 | 20 | 12 | 8 | 7 | 12 |
|  | p: 3 | 0,500 | 0,000 | 0,964 | 0,725 | 0,667 | 0,875 | 0,571 | 0,833 |
|  | p: 4 | 0,500 | 1,000 | 0,036 | 0,275 | 0,333 | 0,125 | 0,429 | 0,167 |
| SNP_012 | N | 10 | 10 | 14 | 17 | 12 | 8 | 7 | 12 |
|  | p: 2 | 1,000 | 1,000 | 0,036 | 0,765 | 1,000 | 1,000 | 0,857 | 0,292 |
|  | p: 4 | 0,000 | 0,000 | 0,964 | 0,235 | 0,000 | 0,000 | 0,143 | 0,708 |
| SNP_013 | N | 10 | 10 | 14 | 17 | 12 | 8 | 7 | 12 |
|  | p: 2 | 0,000 | 0,000 | 0,714 | 0,118 | 0,000 | 0,000 | 0,000 | 0,250 |
|  | p: 4 | 1,000 | 1,000 | 0,286 | 0,882 | 1,000 | 1,000 | 1,000 | 0,750 |
| SNP_014 | N | 10 | 9 | 14 | 20 | 12 | 8 | 7 | 12 |
|  | p: 2 | 0,200 | 0,167 | 0,000 | 0,350 | 0,250 | 0,500 | 0,429 | 0,250 |
|  | p: 4 | 0,800 | 0,833 | 1,000 | 0,650 | 0,750 | 0,500 | 0,571 | 0,750 |
| SNP_015 | N | 10 | 10 | 14 | 16 | 12 | 8 | 7 | 11 |
|  | p: 2 | 0,150 | 0,000 | 0,857 | 0,375 | 0,042 | 0,563 | 0,071 | 0,773 |
|  | p: 4 | 0,850 | 1,000 | 0,143 | 0,625 | 0,958 | 0,438 | 0,929 | 0,227 |
| SNP_016 | N | 10 | 10 | 12 | 20 | 12 | 8 | 7 | 11 |
|  | p: 1 | 0,800 | 1,000 | 0,417 | 0,525 | 0,792 | 0,813 | 0,857 | 0,591 |
|  | p: 3 | 0,200 | 0,000 | 0,583 | 0,475 | 0,208 | 0,188 | 0,143 | 0,409 |
| SNP_017 | N | 10 | 10 | 14 | 19 | 12 | 8 | 7 | 12 |
|  | p: 1 | 0,550 | 0,500 | 0,107 | 0,368 | 0,500 | 0,500 | 0,500 | 0,333 |
|  | p: 2 | 0,450 | 0,500 | 0,893 | 0,632 | 0,500 | 0,500 | 0,500 | 0,667 |
| SNP_018 | N | 10 | 10 | 14 | 19 | 12 | 8 | 7 | 12 |
|  | p: 2 | 0,200 | 0,000 | 0,214 | 0,211 | 0,167 | 0,500 | 0,214 | 0,292 |
|  | p: 3 | 0,800 | 1,000 | 0,786 | 0,789 | 0,833 | 0,500 | 0,786 | 0,708 |
| SNP_019 | N | 10 | 10 | 14 | 18 | 12 | 8 | 7 | 11 |
|  | p: 1 | 0,000 | 0,000 | 0,821 | 0,278 | 0,042 | 0,000 | 0,071 | 0,409 |
|  | p: 3 | 1,000 | 1,000 | 0,179 | 0,722 | 0,958 | 1,000 | 0,929 | 0,591 |
| SNP_020 | N | 10 | 10 | 14 | 20 | 12 | 8 | 7 | 12 |
|  | p: 3 | 0,050 | 0,000 | 0,964 | 0,350 | 0,125 | 0,000 | 0,071 | 0,583 |
|  | p: 4 | 0,950 | 1,000 | 0,036 | 0,650 | 0,875 | 1,000 | 0,929 | 0,417 |
| SNP_021 | N | 10 | 10 | 14 | 17 | 12 | 8 | 7 | 12 |
|  | p: 2 | 0,900 | 0,850 | 1,000 | 0,941 | 0,875 | 1,000 | 0,857 | 0,875 |
|  | p: 4 | 0,100 | 0,150 | 0,000 | 0,059 | 0,125 | 0,000 | 0,143 | 0,125 |
| SNP_022 | N | 10 | 10 | 14 | 17 | 12 | 8 | 7 | 12 |
|  | p: 2 | 0,650 | 1,000 | 0,071 | 0,294 | 0,417 | 0,688 | 0,571 | 0,375 |
|  | p: 4 | 0,350 | 0,000 | 0,929 | 0,706 | 0,583 | 0,313 | 0,429 | 0,625 |
| SNP_023 | N | 8 | 10 | 14 | 17 | 11 | 7 | 6 | 8 |
|  | p: 1 | 1,000 | 1,000 | 0,036 | 0,676 | 1,000 | 0,929 | 0,917 | 0,625 |
|  | p: 3 | 0,000 | 0,000 | 0,964 | 0,324 | 0,000 | 0,071 | 0,083 | 0,375 |
| SNP_024 | N | 10 | 10 | 14 | 18 | 12 | 8 | 6 | 12 |
|  | p: 1 | 0,750 | 0,000 | 1,000 | 0,917 | 0,792 | 1,000 | 0,917 | 0,958 |
|  | p: 4 | 0,250 | 1,000 | 0,000 | 0,083 | 0,208 | 0,000 | 0,083 | 0,042 |
| SNP_025 | N | 10 | 10 | 13 | 16 | 10 | 7 | 6 | 12 |
|  | p: 2 | 0,950 | 1,000 | 0,500 | 0,844 | 0,950 | 1,000 | 0,917 | 0,583 |
|  | p: 4 | 0,050 | 0,000 | 0,500 | 0,156 | 0,050 | 0,000 | 0,083 | 0,417 |
| SNP_026 | N | 10 | 10 | 14 | 18 | 12 | 8 | 6 | 12 |
|  | p: 2 | 0,950 | 0,850 | 1,000 | 0,833 | 0,792 | 0,813 | 0,583 | 1,000 |
|  | p: 4 | 0,050 | 0,150 | 0,000 | 0,167 | 0,208 | 0,188 | 0,417 | 0,000 |
| SNP_027 | N | 10 | 10 | 14 | 19 | 12 | 8 | 7 | 12 |
|  | p: 2 | 1,000 | 1,000 | 0,857 | 1,000 | 1,000 | 1,000 | 1,000 | 0,958 |
|  | p: 4 | 0,000 | 0,000 | 0,143 | 0,000 | 0,000 | 0,000 | 0,000 | 0,042 |
| SNP_028 | N | 10 | 10 | 14 | 19 | 12 | 8 | 7 | 12 |
|  | p: 1 | 0,750 | 0,350 | 1,000 | 0,921 | 0,833 | 0,625 | 0,857 | 0,917 |
|  | p: 3 | 0,250 | 0,650 | 0,000 | 0,079 | 0,167 | 0,375 | 0,143 | 0,083 |
| SNP_029 | N | 10 | 10 | 14 | 19 | 11 | 8 | 7 | 11 |
|  | p: 1 | 0,750 | 0,350 | 1,000 | 0,921 | 0,864 | 0,625 | 0,857 | 0,955 |
|  | p: 3 | 0,250 | 0,650 | 0,000 | 0,079 | 0,136 | 0,375 | 0,143 | 0,045 |
| SNP_030 | N | 10 | 10 | 9 | 17 | 12 | 8 | 7 | 8 |
|  | p: 1 | 1,000 | 1,000 | 0,833 | 1,000 | 1,000 | 1,000 | 1,000 | 1,000 |
|  | p: 3 | 0,000 | 0,000 | 0,167 | 0,000 | 0,000 | 0,000 | 0,000 | 0,000 |
| SNP_031 | N | 10 | 10 | 14 | 19 | 12 | 8 | 7 | 12 |
|  | p: 2 | 1,000 | 1,000 | 0,036 | 0,579 | 1,000 | 1,000 | 1,000 | 0,208 |
|  | p: 3 | 0,000 | 0,000 | 0,964 | 0,421 | 0,000 | 0,000 | 0,000 | 0,792 |
| SNP_032 | N | 10 | 10 | 14 | 17 | 12 | 8 | 7 | 12 |
|  | p: 1 | 0,000 | 0,000 | 0,143 | 0,088 | 0,000 | 0,000 | 0,000 | 0,042 |
|  | p: 2 | 1,000 | 1,000 | 0,857 | 0,912 | 1,000 | 1,000 | 1,000 | 0,958 |
| SNP_033 | N | 10 | 10 | 13 | 17 | 10 | 8 | 7 | 10 |
|  | p: 1 | 1,000 | 0,950 | 0,000 | 0,765 | 1,000 | 1,000 | 0,929 | 0,650 |
|  | p: 3 | 0,000 | 0,050 | 1,000 | 0,235 | 0,000 | 0,000 | 0,071 | 0,350 |
| SNP_034 | N | 10 | 10 | 14 | 19 | 12 | 8 | 7 | 11 |
|  | p: 1 | 1,000 | 1,000 | 0,250 | 0,711 | 0,917 | 1,000 | 1,000 | 0,545 |
|  | p: 3 | 0,000 | 0,000 | 0,750 | 0,289 | 0,083 | 0,000 | 0,000 | 0,455 |
| SNP_035 | N | 8 | 10 | 13 | 16 | 12 | 8 | 7 | 11 |
|  | p: 1 | 0,938 | 1,000 | 0,000 | 0,625 | 0,917 | 0,938 | 0,857 | 0,455 |
|  | p: 3 | 0,063 | 0,000 | 1,000 | 0,375 | 0,083 | 0,063 | 0,143 | 0,545 |
| SNP_036 | N | 9 | 10 | 14 | 17 | 12 | 8 | 7 | 12 |
|  | p: 1 | 1,000 | 1,000 | 1,000 | 0,971 | 1,000 | 1,000 | 1,000 | 0,917 |
|  | p: 3 | 0,000 | 0,000 | 0,000 | 0,029 | 0,000 | 0,000 | 0,000 | 0,083 |
| SNP_037 | N | 10 | 10 | 14 | 17 | 12 | 8 | 6 | 12 |
|  | p: 2 | 0,400 | 0,000 | 0,107 | 0,265 | 0,542 | 0,438 | 0,417 | 0,167 |
|  | p: 3 | 0,600 | 1,000 | 0,893 | 0,735 | 0,458 | 0,563 | 0,583 | 0,833 |
| SNP_038 | N | 10 | 10 | 14 | 20 | 12 | 8 | 7 | 12 |
|  | p: 1 | 0,000 | 0,000 | 0,286 | 0,075 | 0,000 | 0,000 | 0,000 | 0,000 |
|  | p: 4 | 1,000 | 1,000 | 0,714 | 0,925 | 1,000 | 1,000 | 1,000 | 1,000 |
| SNP_039 | N | 10 | 9 | 14 | 17 | 12 | 8 | 7 | 12 |
|  | p: 2 | 0,300 | 0,000 | 0,643 | 0,794 | 0,500 | 0,438 | 0,786 | 0,542 |
|  | p: 4 | 0,700 | 1,000 | 0,357 | 0,206 | 0,500 | 0,563 | 0,214 | 0,458 |
| SNP_040 | N | 10 | 10 | 14 | 18 | 12 | 8 | 7 | 12 |
|  | p: 2 | 1,000 | 1,000 | 0,393 | 0,972 | 0,875 | 1,000 | 0,929 | 0,625 |
|  | p: 4 | 0,000 | 0,000 | 0,607 | 0,028 | 0,125 | 0,000 | 0,071 | 0,375 |
| SNP_041 | N | 10 | 10 | 14 | 18 | 11 | 8 | 7 | 12 |
|  | p: 3 | 0,000 | 0,000 | 0,679 | 0,000 | 0,000 | 0,000 | 0,000 | 0,208 |
|  | p: 4 | 1,000 | 1,000 | 0,321 | 1,000 | 1,000 | 1,000 | 1,000 | 0,792 |
| SNP_042 | N | 10 | 10 | 12 | 15 | 12 | 8 | 7 | 11 |
|  | p: 2 | 0,000 | 0,000 | 0,750 | 0,400 | 0,000 | 0,000 | 0,000 | 0,318 |
|  | p: 4 | 1,000 | 1,000 | 0,250 | 0,600 | 1,000 | 1,000 | 1,000 | 0,682 |
| SNP_043 | N | 10 | 10 | 13 | 17 | 12 | 8 | 7 | 12 |
|  | p: 1 | 0,850 | 1,000 | 0,000 | 0,529 | 0,875 | 0,938 | 0,929 | 0,208 |
|  | p: 3 | 0,150 | 0,000 | 1,000 | 0,471 | 0,125 | 0,063 | 0,071 | 0,792 |
| SNP_044 | N | 10 | 9 | 14 | 14 | 10 | 8 | 7 | 12 |
|  | p: 2 | 0,550 | 0,000 | 1,000 | 0,786 | 0,800 | 0,875 | 0,357 | 0,667 |
|  | p: 4 | 0,450 | 1,000 | 0,000 | 0,214 | 0,200 | 0,125 | 0,643 | 0,333 |
| SNP_045 | N | 10 | 10 | 14 | 19 | 12 | 8 | 7 | 12 |
|  | p: 1 | 0,000 | 0,000 | 0,393 | 0,079 | 0,042 | 0,000 | 0,000 | 0,167 |
|  | p: 3 | 1,000 | 1,000 | 0,607 | 0,921 | 0,958 | 1,000 | 1,000 | 0,833 |
| SNP_046 | N | 10 | 10 | 14 | 17 | 12 | 8 | 7 | 12 |
|  | p: 2 | 1,000 | 1,000 | 0,679 | 0,971 | 1,000 | 1,000 | 1,000 | 0,667 |
|  | p: 4 | 0,000 | 0,000 | 0,321 | 0,029 | 0,000 | 0,000 | 0,000 | 0,333 |
| SNP_047 | N | 10 | 10 | 14 | 18 | 12 | 8 | 7 | 12 |
|  | p: 2 | 0,050 | 0,000 | 0,786 | 0,250 | 0,000 | 0,000 | 0,000 | 0,417 |
|  | p: 3 | 0,950 | 1,000 | 0,214 | 0,750 | 1,000 | 1,000 | 1,000 | 0,583 |
| SNP_048 | N | 10 | 10 | 14 | 20 | 12 | 8 | 7 | 12 |
|  | p: 2 | 0,500 | 0,500 | 0,429 | 0,425 | 0,500 | 0,500 | 0,500 | 0,417 |
|  | p: 4 | 0,500 | 0,500 | 0,571 | 0,575 | 0,500 | 0,500 | 0,500 | 0,583 |
| SNP_049 | N | 10 | 10 | 14 | 19 | 12 | 8 | 7 | 12 |
|  | p: 3 | 1,000 | 1,000 | 0,000 | 0,605 | 0,958 | 1,000 | 1,000 | 0,292 |
|  | p: 4 | 0,000 | 0,000 | 1,000 | 0,395 | 0,042 | 0,000 | 0,000 | 0,708 |
| SNP_050 | N | 10 | 10 | 14 | 18 | 12 | 8 | 7 | 12 |
|  | p: 2 | 0,000 | 0,000 | 0,714 | 0,389 | 0,083 | 0,000 | 0,000 | 0,375 |
|  | p: 4 | 1,000 | 1,000 | 0,286 | 0,611 | 0,917 | 1,000 | 1,000 | 0,625 |
| SNP_051 | N | 10 | 10 | 14 | 18 | 12 | 8 | 7 | 12 |
|  | p: 1 | 0,000 | 0,000 | 0,857 | 0,528 | 0,042 | 0,000 | 0,000 | 0,625 |
|  | p: 3 | 1,000 | 1,000 | 0,143 | 0,472 | 0,958 | 1,000 | 1,000 | 0,375 |
| SNP_052 | N | 10 | 10 | 13 | 18 | 12 | 8 | 7 | 12 |
|  | p: 1 | 0,000 | 0,000 | 0,923 | 0,611 | 0,042 | 0,000 | 0,000 | 0,458 |
|  | p: 3 | 1,000 | 1,000 | 0,077 | 0,389 | 0,958 | 1,000 | 1,000 | 0,542 |
| SNP_053 | N | 10 | 10 | 14 | 20 | 12 | 8 | 7 | 12 |
|  | p: 2 | 0,400 | 0,050 | 1,000 | 0,450 | 0,167 | 0,500 | 0,357 | 0,750 |
|  | p: 4 | 0,600 | 0,950 | 0,000 | 0,550 | 0,833 | 0,500 | 0,643 | 0,250 |
| SNP_054 | N | 10 | 10 | 14 | 17 | 12 | 8 | 7 | 12 |
|  | p: 2 | 0,050 | 0,000 | 0,786 | 0,735 | 0,292 | 0,063 | 0,143 | 0,708 |
|  | p: 4 | 0,950 | 1,000 | 0,214 | 0,265 | 0,708 | 0,938 | 0,857 | 0,292 |
| SNP_055 | N | 10 | 10 | 14 | 18 | 12 | 8 | 7 | 12 |
|  | p: 1 | 0,650 | 0,950 | 0,000 | 0,278 | 0,542 | 0,188 | 0,571 | 0,250 |
|  | p: 2 | 0,350 | 0,050 | 1,000 | 0,722 | 0,458 | 0,813 | 0,429 | 0,750 |
| SNP_056 | N | 9 | 10 | 14 | 18 | 12 | 8 | 7 | 12 |
|  | p: 1 | 0,889 | 0,950 | 1,000 | 0,472 | 0,208 | 0,000 | 0,000 | 0,625 |
|  | p: 3 | 0,111 | 0,050 | 0,000 | 0,528 | 0,792 | 1,000 | 1,000 | 0,375 |
| SNP_057 | N | 10 | 10 | 13 | 17 | 12 | 8 | 6 | 11 |
|  | p: 2 | 0,000 | 0,000 | 0,962 | 0,647 | 0,000 | 0,000 | 0,000 | 0,636 |
|  | p: 4 | 1,000 | 1,000 | 0,038 | 0,353 | 1,000 | 1,000 | 1,000 | 0,364 |
| SNP_058 | N | 10 | 10 | 12 | 19 | 11 | 8 | 7 | 12 |
|  | p: 1 | 1,000 | 0,950 | 1,000 | 1,000 | 1,000 | 1,000 | 1,000 | 0,875 |
|  | p: 3 | 0,000 | 0,050 | 0,000 | 0,000 | 0,000 | 0,000 | 0,000 | 0,125 |
| SNP_059 | N | 9 | 9 | 14 | 16 | 12 | 8 | 7 | 12 |
|  | p: 1 | 0,056 | 0,111 | 0,000 | 0,063 | 0,208 | 0,125 | 0,357 | 0,000 |
|  | p: 4 | 0,944 | 0,889 | 1,000 | 0,938 | 0,792 | 0,875 | 0,643 | 1,000 |
| SNP_060 | N | 10 | 10 | 14 | 18 | 12 | 8 | 6 | 12 |
|  | p: 1 | 0,600 | 0,500 | 1,000 | 0,556 | 0,375 | 0,250 | 0,083 | 0,667 |
|  | p: 3 | 0,400 | 0,500 | 0,000 | 0,444 | 0,625 | 0,750 | 0,917 | 0,333 |
| SNP_061 | N | 10 | 10 | 14 | 17 | 12 | 8 | 7 | 12 |
|  | p: 2 | 1,000 | 0,800 | 1,000 | 0,882 | 0,792 | 1,000 | 1,000 | 1,000 |
|  | p: 4 | 0,000 | 0,200 | 0,000 | 0,118 | 0,208 | 0,000 | 0,000 | 0,000 |
| SNP_062 | N | 10 | 10 | 14 | 19 | 12 | 8 | 7 | 12 |
|  | p: 2 | 0,450 | 0,800 | 0,964 | 0,895 | 0,417 | 0,438 | 0,500 | 0,875 |
|  | p: 3 | 0,550 | 0,200 | 0,036 | 0,105 | 0,583 | 0,563 | 0,500 | 0,125 |
| SNP_063 | N | 10 | 10 | 14 | 20 | 12 | 8 | 7 | 12 |
|  | p: 1 | 0,050 | 0,000 | 0,000 | 0,275 | 0,292 | 0,188 | 0,571 | 0,000 |
|  | p: 3 | 0,950 | 1,000 | 1,000 | 0,725 | 0,708 | 0,813 | 0,429 | 1,000 |
| SNP_064 | N | 10 | 10 | 14 | 17 | 11 | 8 | 6 | 11 |
|  | p: 1 | 0,400 | 0,300 | 0,000 | 0,088 | 0,455 | 0,375 | 0,417 | 0,136 |
|  | p: 2 | 0,600 | 0,700 | 1,000 | 0,912 | 0,545 | 0,625 | 0,583 | 0,864 |
| SNP_065 | N | 5 | 7 | 13 | 17 | 12 | 8 | 7 | 8 |
|  | p: 3 | 0,200 | 0,143 | 1,000 | 0,147 | 0,000 | 0,125 | 0,000 | 0,500 |
|  | p: 4 | 0,800 | 0,857 | 0,000 | 0,853 | 1,000 | 0,875 | 1,000 | 0,500 |
| SNP_066 | N | 5 | 10 | 14 | 16 | 10 | 5 | 2 | 12 |
|  | p: 1 | 1,000 | 1,000 | 1,000 | 0,969 | 0,750 | 0,700 | 0,500 | 0,750 |
|  | p: 3 | 0,000 | 0,000 | 0,000 | 0,031 | 0,250 | 0,300 | 0,500 | 0,250 |
| SNP_067 | N | 8 | 10 | 12 | 18 | 12 | 8 | 6 | 11 |
|  | p: 1 | 0,063 | 0,000 | 0,083 | 0,000 | 0,125 | 0,000 | 0,000 | 0,227 |
|  | p: 3 | 0,938 | 1,000 | 0,917 | 1,000 | 0,875 | 1,000 | 1,000 | 0,773 |
| SNP_068 | N | 10 | 10 | 14 | 17 | 12 | 8 | 7 | 12 |
|  | p: 1 | 0,700 | 0,950 | 1,000 | 0,735 | 0,458 | 0,375 | 0,571 | 0,625 |
|  | p: 2 | 0,300 | 0,050 | 0,000 | 0,265 | 0,542 | 0,625 | 0,429 | 0,375 |
| SNP_069 | N | 9 | 10 | 14 | 16 | 12 | 8 | 6 | 12 |
|  | p: 4 | 1,000 | 1,000 | 1,000 | 1,000 | 1,000 | 1,000 | 1,000 | 1,000 |
|  |  |  |  |  |  |  |  |  |  |
| SNP_070 | N | 10 | 9 | 14 | 18 | 12 | 8 | 6 | 12 |
|  | p: 2 | 0,600 | 0,889 | 0,929 | 0,778 | 0,417 | 0,250 | 0,250 | 0,833 |
|  | p: 4 | 0,400 | 0,111 | 0,071 | 0,222 | 0,583 | 0,750 | 0,750 | 0,167 |
| SNP_071 | N | 10 | 10 | 14 | 20 | 12 | 8 | 7 | 12 |
|  | p: 1 | 0,200 | 0,000 | 0,036 | 0,225 | 0,292 | 0,500 | 0,500 | 0,042 |
|  | p: 3 | 0,800 | 1,000 | 0,964 | 0,775 | 0,708 | 0,500 | 0,500 | 0,958 |
| SNP_072 | N | 9 | 10 | 13 | 19 | 12 | 8 | 6 | 12 |
|  | p: 2 | 0,556 | 0,050 | 0,038 | 0,132 | 0,167 | 0,375 | 0,083 | 0,208 |
|  | p: 4 | 0,444 | 0,950 | 0,962 | 0,868 | 0,833 | 0,625 | 0,917 | 0,792 |
| SNP_073 | N | 10 | 10 | 14 | 16 | 12 | 6 | 7 | 12 |
|  | p: 2 | 0,700 | 0,350 | 0,500 | 0,500 | 0,542 | 0,583 | 0,500 | 0,500 |
|  | p: 4 | 0,300 | 0,650 | 0,500 | 0,500 | 0,458 | 0,417 | 0,500 | 0,500 |
| SNP_074 | N | 10 | 10 | 14 | 18 | 12 | 8 | 7 | 12 |
|  | p: 1 | 0,850 | 1,000 | 0,893 | 0,944 | 0,875 | 0,938 | 1,000 | 0,917 |
|  | p: 3 | 0,150 | 0,000 | 0,107 | 0,056 | 0,125 | 0,063 | 0,000 | 0,083 |
| SNP_075 | N | 10 | 10 | 14 | 19 | 12 | 8 | 7 | 12 |
|  | p: 2 | 0,900 | 1,000 | 0,821 | 0,842 | 0,708 | 1,000 | 0,786 | 1,000 |
|  | p: 4 | 0,100 | 0,000 | 0,179 | 0,158 | 0,292 | 0,000 | 0,214 | 0,000 |
| SNP_076 | N | 10 | 10 | 14 | 19 | 12 | 8 | 7 | 12 |
|  | p: 2 | 0,050 | 0,000 | 0,143 | 0,184 | 0,125 | 0,000 | 0,071 | 0,042 |
|  | p: 4 | 0,950 | 1,000 | 0,857 | 0,816 | 0,875 | 1,000 | 0,929 | 0,958 |
| SNP_077 | N | 10 | 10 | 14 | 20 | 12 | 8 | 7 | 12 |
|  | p: 2 | 0,100 | 0,250 | 0,000 | 0,000 | 0,000 | 0,000 | 0,000 | 0,083 |
|  | p: 4 | 0,900 | 0,750 | 1,000 | 1,000 | 1,000 | 1,000 | 1,000 | 0,917 |
| SNP_078 | N | 10 | 10 | 14 | 17 | 12 | 8 | 7 | 12 |
|  | p: 2 | 1,000 | 0,700 | 0,964 | 0,912 | 0,750 | 1,000 | 1,000 | 0,875 |
|  | p: 3 | 0,000 | 0,300 | 0,036 | 0,088 | 0,250 | 0,000 | 0,000 | 0,125 |
| SNP_079 | N | 9 | 10 | 14 | 17 | 12 | 7 | 7 | 12 |
|  | p: 2 | 0,722 | 0,850 | 1,000 | 0,735 | 0,625 | 0,714 | 0,286 | 0,917 |
|  | p: 4 | 0,278 | 0,150 | 0,000 | 0,265 | 0,375 | 0,286 | 0,714 | 0,083 |
| SNP_080 | N | 10 | 10 | 14 | 19 | 12 | 8 | 7 | 12 |
|  | p: 3 | 0,050 | 0,050 | 0,250 | 0,368 | 0,292 | 0,313 | 0,357 | 0,333 |
|  | p: 4 | 0,950 | 0,950 | 0,750 | 0,632 | 0,708 | 0,688 | 0,643 | 0,667 |
| SNP_081 | N | 10 | 10 | 14 | 17 | 12 | 8 | 7 | 12 |
|  | p: 2 | 0,500 | 0,100 | 0,000 | 0,235 | 0,417 | 0,375 | 0,429 | 0,042 |
|  | p: 4 | 0,500 | 0,900 | 1,000 | 0,765 | 0,583 | 0,625 | 0,571 | 0,958 |
| SNP_082 | N | 9 | 8 | 14 | 19 | 10 | 8 | 7 | 11 |
|  | p: 3 | 1,000 | 0,938 | 1,000 | 0,974 | 1,000 | 1,000 | 1,000 | 1,000 |
|  | p: 4 | 0,000 | 0,063 | 0,000 | 0,026 | 0,000 | 0,000 | 0,000 | 0,000 |
| SNP_083 | N | 9 | 10 | 14 | 18 | 12 | 8 | 7 | 11 |
|  | p: 1 | 0,667 | 1,000 | 0,893 | 0,889 | 0,917 | 0,750 | 0,929 | 0,864 |
|  | p: 3 | 0,333 | 0,000 | 0,107 | 0,111 | 0,083 | 0,250 | 0,071 | 0,136 |
| SNP_084 | N | 10 | 10 | 14 | 16 | 12 | 8 | 7 | 12 |
|  | p: 3 | 0,550 | 0,000 | 0,000 | 0,719 | 0,583 | 0,625 | 0,857 | 0,167 |
|  | p: 4 | 0,450 | 1,000 | 1,000 | 0,281 | 0,417 | 0,375 | 0,143 | 0,833 |
| SNP_085 | N | 10 | 10 | 14 | 19 | 12 | 8 | 7 | 12 |
|  | p: 2 | 0,550 | 0,950 | 1,000 | 0,711 | 0,625 | 0,188 | 0,571 | 0,875 |
|  | p: 3 | 0,450 | 0,050 | 0,000 | 0,289 | 0,375 | 0,813 | 0,429 | 0,125 |
| SNP_086 | N | 10 | 10 | 13 | 19 | 12 | 7 | 5 | 9 |
|  | p: 2 | 0,750 | 0,300 | 0,000 | 0,816 | 0,958 | 0,929 | 1,000 | 0,556 |
|  | p: 4 | 0,250 | 0,700 | 1,000 | 0,184 | 0,042 | 0,071 | 0,000 | 0,444 |
| SNP_087 | N | 10 | 10 | 14 | 17 | 12 | 7 | 7 | 12 |
|  | p: 1 | 0,100 | 0,000 | 0,143 | 0,265 | 0,167 | 0,071 | 0,000 | 0,208 |
|  | p: 3 | 0,900 | 1,000 | 0,857 | 0,735 | 0,833 | 0,929 | 1,000 | 0,792 |
| SNP_088 | N | 10 | 10 | 14 | 16 | 12 | 8 | 7 | 12 |
|  | p: 2 | 0,950 | 0,750 | 1,000 | 0,906 | 0,875 | 1,000 | 0,571 | 1,000 |
|  | p: 4 | 0,050 | 0,250 | 0,000 | 0,094 | 0,125 | 0,000 | 0,429 | 0,000 |
| SNP_089 | N | 10 | 10 | 14 | 20 | 12 | 8 | 7 | 12 |
|  | p: 2 | 0,850 | 0,950 | 1,000 | 0,950 | 0,833 | 0,813 | 0,714 | 0,958 |
|  | p: 4 | 0,150 | 0,050 | 0,000 | 0,050 | 0,167 | 0,188 | 0,286 | 0,042 |
| SNP_090 | N | 10 | 10 | 14 | 18 | 12 | 8 | 7 | 12 |
|  | p: 2 | 0,150 | 0,000 | 0,000 | 0,194 | 0,250 | 0,500 | 0,143 | 0,000 |
|  | p: 4 | 0,850 | 1,000 | 1,000 | 0,806 | 0,750 | 0,500 | 0,857 | 1,000 |
| SNP_091 | N | 8 | 9 | 13 | 13 | 9 | 7 | 6 | 10 |
|  | p: 1 | 0,250 | 0,222 | 0,000 | 0,462 | 0,444 | 0,429 | 0,417 | 0,200 |
|  | p: 3 | 0,750 | 0,778 | 1,000 | 0,538 | 0,556 | 0,571 | 0,583 | 0,800 |
| SNP_092 | N | 10 | 10 | 14 | 17 | 11 | 8 | 6 | 11 |
|  | p: 2 | 0,700 | 0,950 | 1,000 | 0,971 | 0,636 | 0,875 | 0,667 | 0,818 |
|  | p: 4 | 0,300 | 0,050 | 0,000 | 0,029 | 0,364 | 0,125 | 0,333 | 0,182 |
| SNP_093 | N | 10 | 10 | 14 | 19 | 12 | 8 | 7 | 12 |
|  | p: 1 | 0,200 | 0,100 | 0,071 | 0,211 | 0,333 | 0,375 | 0,357 | 0,208 |
|  | p: 3 | 0,800 | 0,900 | 0,929 | 0,789 | 0,667 | 0,625 | 0,643 | 0,792 |
| SNP_094 | N | 10 | 10 | 14 | 19 | 12 | 8 | 7 | 12 |
|  | p: 2 | 0,600 | 0,800 | 1,000 | 0,632 | 0,667 | 0,750 | 0,643 | 0,917 |
|  | p: 4 | 0,400 | 0,200 | 0,000 | 0,368 | 0,333 | 0,250 | 0,357 | 0,083 |
| SNP_095 | N | 10 | 10 | 14 | 18 | 12 | 8 | 7 | 11 |
|  | p: 1 | 0,500 | 0,050 | 0,429 | 0,444 | 0,542 | 0,375 | 0,714 | 0,500 |
|  | p: 3 | 0,500 | 0,950 | 0,571 | 0,556 | 0,458 | 0,625 | 0,286 | 0,500 |
| SNP_096 | N | 10 | 8 | 14 | 17 | 12 | 8 | 7 | 12 |
|  | p: 1 | 0,300 | 0,000 | 0,000 | 0,353 | 0,542 | 0,438 | 0,214 | 0,042 |
|  | p: 3 | 0,700 | 1,000 | 1,000 | 0,647 | 0,458 | 0,563 | 0,786 | 0,958 |
| SNP_097 | N | 10 | 10 | 14 | 19 | 12 | 8 | 7 | 12 |
|  | p: 2 | 0,050 | 0,000 | 0,571 | 0,368 | 0,292 | 0,438 | 0,071 | 0,250 |
|  | p: 4 | 0,950 | 1,000 | 0,429 | 0,632 | 0,708 | 0,563 | 0,929 | 0,750 |
| SNP_098 | N | 8 | 10 | 14 | 17 | 11 | 5 | 4 | 11 |
|  | p: 2 | 0,875 | 0,800 | 1,000 | 0,971 | 0,909 | 0,900 | 0,750 | 0,773 |
|  | p: 4 | 0,125 | 0,200 | 0,000 | 0,029 | 0,091 | 0,100 | 0,250 | 0,227 |
| SNP_099 | N | 10 | 10 | 14 | 16 | 12 | 8 | 6 | 12 |
|  | p: 2 | 1,000 | 1,000 | 1,000 | 1,000 | 1,000 | 1,000 | 1,000 | 1,000 |
|  |  |  |  |  |  |  |  |  |  |
| SNP_100 | N | 10 | 10 | 14 | 17 | 11 | 8 | 7 | 11 |
|  | p: 1 | 0,350 | 0,400 | 0,286 | 0,324 | 0,318 | 0,500 | 0,143 | 0,409 |
|  | p: 2 | 0,650 | 0,600 | 0,714 | 0,676 | 0,682 | 0,500 | 0,857 | 0,591 |
| SNP_101 | N | 10 | 10 | 14 | 20 | 12 | 8 | 7 | 12 |
|  | p: 2 | 0,250 | 0,050 | 0,000 | 0,050 | 0,292 | 0,750 | 0,071 | 0,125 |
|  | p: 3 | 0,750 | 0,950 | 1,000 | 0,950 | 0,708 | 0,250 | 0,929 | 0,875 |
| SNP_102 | N | 10 | 10 | 13 | 13 | 9 | 8 | 7 | 9 |
|  | p: 1 | 0,900 | 1,000 | 0,923 | 0,731 | 0,722 | 0,813 | 0,714 | 0,833 |
|  | p: 2 | 0,100 | 0,000 | 0,077 | 0,269 | 0,278 | 0,188 | 0,286 | 0,167 |
| SNP_103 | N | 10 | 10 | 14 | 18 | 12 | 7 | 6 | 12 |
|  | p: 3 | 0,500 | 0,950 | 1,000 | 0,667 | 0,542 | 0,286 | 0,583 | 0,958 |
|  | p: 4 | 0,500 | 0,050 | 0,000 | 0,333 | 0,458 | 0,714 | 0,417 | 0,042 |
| SNP_104 | N | 10 | 9 | 8 | 13 | 12 | 7 | 6 | 7 |
|  | p: 2 | 0,850 | 1,000 | 0,250 | 0,846 | 0,958 | 0,929 | 0,917 | 0,429 |
|  | p: 4 | 0,150 | 0,000 | 0,750 | 0,154 | 0,042 | 0,071 | 0,083 | 0,571 |
| SNP_105 | N | 10 | 10 | 14 | 17 | 12 | 8 | 7 | 11 |
|  | p: 2 | 0,100 | 0,000 | 0,000 | 0,000 | 0,083 | 0,000 | 0,071 | 0,045 |
|  | p: 4 | 0,900 | 1,000 | 1,000 | 1,000 | 0,917 | 1,000 | 0,929 | 0,955 |
| SNP_106 | N | 10 | 7 | 14 | 16 | 12 | 8 | 7 | 12 |
|  | p: 1 | 0,900 | 0,857 | 1,000 | 1,000 | 0,958 | 0,938 | 1,000 | 1,000 |
|  | p: 3 | 0,100 | 0,143 | 0,000 | 0,000 | 0,042 | 0,063 | 0,000 | 0,000 |
