# Supplementary material for: Transcriptome-Based SNP Discovery and Validation in the Hybrid Zone of the Neotropical Annual Fish Genus Austrolebias
Source: Genes (Basel). 2019 Oct 11;10(10):789. doi: 10.3390/genes10100789 (PMC6826752; doi:10.3390/genes10100789)
Supplement: Supplementary file 1 [file genes-10-00789-s001.zip › genes-572550-supplementary-proof/Table S5 Departure from HWE.docx]

**Table S5**

Departure of the Hardy Weinberg Equilibrium (HWE) at some loci in the 8 populations of the hybrid zone from DMS in South America. P < 0.05. s.d.: standard deviation.

| **Population** | **Locus** | **P-value** | **s.d.** |
| --- | --- | --- | --- |
| CH66 | SNP_005 | 0.052 | 0.000 |
|  | SNP_017 | 0.007 | 0.000 |
|  | SNP_048 | 0.007 | 0.000 |
|  | SNP_088 | 0.050 | 0.000 |
| CH64 | SNP_006 | 0.012 | 0.000 |
|  | SNP_044 | 0.015 | 0.000 |
|  | SNP_083 | 0.005 | 0.000 |
| CH43 | SNP_013 | 0.000 | 0.000 |
|  | SNP_018 | 0.000 | 0.000 |
|  | SNP_034 | 0.006 | 0.000 |
|  | SNP_048 | 0.021 | 0.000 |
|  | SNP_073 | 0.000 | 0.000 |
|  | SNP_087 | 0.004 | 0.000 |
|  | SNP_102 | 0.040 | 0.000 |
|  | SNP_104 | 0.015 | 0.000 |
| CH60 | SNP_005 | 0.002 | 0.000 |
|  | SNP_006 | 0.002 | 0.000 |
|  | SNP_013 | 0.002 | 0.000 |
|  | SNP_017 | 0.040 | 0.000 |
|  | SNP_025 | 0.020 | 0.000 |
|  | SNP_044 | 0.000 | 0.000 |
|  | SNP_065 | 0.014 | 0.000 |
|  | SNP_073 | 0.000 | 0.000 |
|  | SNP_074 | 0.030 | 0.000 |
|  | SNP_083 | 0.002 | 0.000 |
|  | SNP_087 | 0.040 | 0.000 |
|  | SNP_090 | 0.003 | 0.000 |
|  | SNP_095 | 0.022 | 0.000 |
| CH54-61 | SNP_006 | 0.050 | 0.000 |
|  | SNP_017 | 0.001 | 0.000 |
|  | SNP_048 | 0.001 | 0.000 |
|  | SNP_059 | 0.031 | 0.000 |
|  | SNP_064 | 0.020 | 0.000 |
| CHN3 | SNP_017 | 0.030 | 0.000 |
|  | SNP_048 | 0.030 | 0.000 |
|  | SNP_083 | 0.020 | 0.000 |
|  | SNP_090 | 0.005 | 0.000 |
| CHN4 | SNP_017 | 0.040 | 0.000 |
|  | SNP_048 | 0.040 | 0.000 |
| CHN6 | SNP_013 | 0.001 | 0.000 |
|  | SNP_023 | 0.006 | 0.000 |
|  | SNP_044 | 0.043 | 0.000 |
|  | SNP_066 | 0.001 | 0.000 |
|  | SNP_073 | 0.001 | 0.000 |
|  | SNP_074 | 0.043 | 0.000 |
|  | SNP_086 | 0.002 | 0.000 |
|  | SNP_087 | 0.031 | 0.000 |
|  | SNP_092 | 0.007 | 0.000 |
|  | SNP_104 | 0.011 | 0.000 |
