# Supplementary material for: Transcriptome-Based SNP Discovery and Validation in the Hybrid Zone of the Neotropical Annual Fish Genus Austrolebias
Source: Genes (Basel). 2019 Oct 11;10(10):789. doi: 10.3390/genes10100789 (PMC6826752; doi:10.3390/genes10100789)
Supplement: Supplementary file 1 [file genes-10-00789-s001.zip › genes-572550-supplementary-proof/Table S6 Cytb.docx]

**Table S6**

Pairwise K2P sequence distances based on Cytb in 8 populations (below the diagonal) from DMS in South America (above diagonal, standard deviation). Significant values (P=0.05).

|  |  | **1** | **2** | **3** | **4** | **5** | **6** | **7** | **8** |
| --- | --- | --- | --- | --- | --- | --- | --- | --- | --- |
| **1.** | **CH66** |  | 0.002 | 0.002 | 0.003 | 0.003 | 0.009 | 0.003 | 0.012 |
| **2.** | **CH64** | 0.006 |  | 0.001 | 0.002 | 0.002 | 0.009 | 0.002 | 0.012 |
| **3.** | **CHN3** | 0.006 | 0.002 |  | 0.003 | 0.002 | 0.009 | 0.002 | 0.012 |
| **4.** | **CHN4** | 0.010 | 0.007 | 0.007 |  | 0.003 | 0.009 | 0.002 | 0.012 |
| **5.** | **CH60** | 0.021 | 0.017 | 0.018 | 0.020 |  | 0.008 | 0.003 | 0.010 |
| **6.** | **CHN6** | 0.070 | 0.065 | 0.066 | 0.067 | 0.059 |  | 0.009 | 0.003 |
| **7.** | **CH54-61** | 0.010 | 0.007 | 0.008 | 0.005 | 0.019 | 0.066 |  | 0.012 |
| **8.** | **CH43** | 0.091 | 0.086 | 0.087 | 0.089 | 0.073 | 0.026 | 0.088 |  |
